# A multilevel network approach to revealing patterns of online political selective exposure

**DOI:** 10.1371/journal.pone.0332663

**Published:** 2025-09-22

**Authors:** Yuan Zhang, Laia Castro, Frank Esser, Alexandre Bovet

**Affiliations:** 1 Department of Communication and Media Research, University of Zurich, Zurich, Switzerland; 2 Department of Political Science, University of Barcelona, Barcelona, Spain; 3 Department of Mathematical Modeling and Machine Learning, University of Zurich, Zurich, Switzerland; 4 Digital Society Initiative, University of Zurich, Zurich, Switzerland; Universidade de Santiago de Compostela, SPAIN

## Abstract

Selective exposure, individuals’ inclination to seek out information that supports their beliefs while avoiding information that contradicts them, plays an important role in the emergence of polarization and echo chambers. In the political domain, selective exposure is usually measured on a left-right ideology scale, ignoring finer details. To bridge the gap, this work introduces a multilevel analysis framework based on a multi-scale community detection approach. To test this approach, we combine survey and Twitter/X data collected during the 2022 Brazilian Presidential Election and investigate selective exposure patterns among survey respondents in their choices of whom to follow. We construct a bipartite network connecting survey respondents with political influencers and project it onto the influencer nodes. Applying multi-scale community detection to this projection uncovers a hierarchical clustering of political influencers, where each cluster is more frequently co-followed by a specific subgroup of survey respondents compared to others. Different indices of selective exposure, such as Community Overlap, Identity Diversity, Information Diversity, Structural Integration, and Connectivity Inequality, suggest that the characteristics of the influencer communities engaged by survey respondents vary with the level of community resolution. This finding indicates that online political selective exposure exhibits a more complex structure than a mere left-right dichotomy. Moreover, depending on the resolution level we consider, we find different associations between network indices of exposure patterns and 189 individual attributes of the survey respondents. For example, at finer levels, survey respondents’ Community Overlap is associated with several factors, such as ideological position, demographics, news consumption frequency, and incivility perception. In comparison, only their ideological position is a significant factor at coarser levels. Our work demonstrates that measuring selective exposure at a single level, such as left and right, misses important information necessary to capture this phenomenon correctly.

## Introduction

The internet, especially social media, has facilitated the online consumption of political information in recent decades [1]. According to a national survey we conducted during the 2022 Brazilian Presidential Election, 82 percent of the Brazilian population acquired political information via social media, with approximately 51 percent of them consuming or engaging with political information on Twitter (now known as X) at least once a week. On Twitter/X, ordinary users mainly receive political information by following certain political influencers, such as politicians, media outlets, journalists, and individual opinion leaders. Despite social networks like Twitter/X providing a high-choice environment for information consumption and the possibility of deliberative communication, it is a concern that users on social networks predominantly follow information aligned with their own opinions, leading to political fragmentation, polarization, and the creation of echo chambers [2–6]. This phenomenon, known as selective exposure, is attributed to bounded rationality—humans have limited capacity for information processing and prefer to be exposed to information they find agreeable [7] and to the influence of personalized recommendation algorithms on social media platforms [8–13]. Selective exposure online can, therefore, limit people’s access to diverse opinions and even lead to the spread of extreme views within certain groups [14].

Many studies have explored selective exposure and related topics such as political polarization and echo chambers, e.g., [5,15–22]. Most of them focus on the U.S. case, where the political system has traditionally been divided between two major parties - the Democrats and the Republicans [23]. A similar left–right division is evident in users’ behaviorally selective exposure, where individuals are more likely to interact with members of their own party [24]. This phenomenon deepens polarization both ideologically and behaviorally through the reinforcement of echo chambers [25]. However, the dichotomous model of selective exposure, polarization, or echo chambers might not be fully applicable to countries like Brazil, which has a multi-party system and weak party affiliation. Scholars often observe non-ideological cleavage fragmentation in multi-party systems, although the left–right ideological divide persists [26]. Therefore, this study focuses on Brazil as a case study, where both non-ideological fragmentation and ideological division may contribute to the complex patterns of selective exposure among Brazilian online users.

Furthermore, user-generated content on social media has reshaped the dichotomous nature of online political dynamics globally [27–29]. It is not only elite accounts such as media outlets, political parties, and politicians that promote political propaganda, but also individual opinion leaders who create more targeted political information to represent specific social groups or achieve business attention [30,31]. In light of this background, political division is more complex in the contemporary era than ever. Evidence from the 2016 U.S. presidential election presented by Bartels *et al.* [33] highlights that within the Democratic group, various cultural subgroups exist where social identities and other demographic characteristics play a role in forming closely connected communities. Similarly, Brazil’s party systems are also intertwined with complex socio-cultural groups that could even transcend party lines [32].

That said, traditional network community detection algorithms that perform well in identifying left–right selective exposure, polarization, and echo chambers in the U.S. are often inadequate for uncovering fragmentation patterns in countries like Brazil or in the current social media age. Modularity-based quality functions suffer from a resolution limit, making them ineffective at detecting smaller communities within large networks and at multiple resolution levels [34]. Hence, in this study, we apply a multi-scale community detection framework based on Markov stability to uncover patterns of political selective exposure among Brazilian online users sampled from a representative national survey across varying levels of resolution [35]. While other network-based approaches can also measure polarization in multipolar online systems, our approach offers a distinct advantage in uncovering the hierarchical structure of political systems [61,62]. Additionally, we propose five novel indices of selective exposure and examine their associated factors at various resolution levels. For each surveyed individual, they are computed based on the communities that their followed influencers form. They are: (i) *Community Overlap*, capturing the bridging capacity of the surveyed individuals; (ii) *Identity Diversity*, measuring the diversity of the identities of influencers that surveyed individuals follow; (iii) *Information Diversity*, capturing the information provision diversity of their influencers; (iv) *Structural Integration*, measuring the network integration of their influencers; and (v) *Connectivity Inequality*, capturing the influence imbalance of their influencers. Overall, our research is guided by two main questions:

RQ1: How can we capture the complexity of individuals’ selective exposure, reflected in Community Overlap, Identity Diversity, Information Diversity, Structural Integration, and Connectivity Inequality, within a fragmented political context?
RQ2: Which individual attributes are associated with selective exposure patterns across different resolution levels?

To answer the two research questions, we combine a representative national survey and Twitter/X political influencers the survey participants followed, and construct consumer-supplier relations. Consumers are represented by 204 respondents from a national survey who provided their Twitter/X handles, while the suppliers are 2,307 political influencers (including politicians, media, and individual influencers) followed by consumers. The distribution of demographic attributes of the 204 survey participants does not show significant statistical differences compared to the overall survey sample, which is representatively sampled from Brazil’s population. Our analysis, therefore, reports on selective exposure from the point of view of a representative sample of users. Moreover, political influencers are annotated based on dimensions of political ideology (Left/Right/Center), campaign support (Lula camp/Bolsonaro camp), social identity (Women/Religious/LGBTQ/Black), and account type (Politician/Media/Individual). Then, we uncover the complex structures of Brazilian users’ selective exposure on Twitter/X using a multi-scale community detection approach. The results suggest that ordinary users tend to expose themselves to political influencers based on ideological positions, however, selective exposure patterns are also evident along other dimensions, such as electoral supports, social identities, and categories of accounts. Therefore, Brazilian users’ selective exposure on Twitter/X forms a multi-level and hierarchical structure.

By examining a comprehensive set of individuals’ survey attributes with the five selective exposure indices at various levels, we find that, at the lowest level, a wide range of variables, including ideological position, demographics, news consumption frequency, and incivility perception, are significantly associated with the survey individuals’ Community Overlap. As the level increases, the ideological position becomes more prominent. The other indices are also associated with different individual attributes at various levels. These findings suggest that the multilevel framework based on multi-scale community detection is an effective approach for revealing individuals’ selective exposure across different levels of resolution, and it can be also applied to other contexts and larger datasets.

This study significantly contributes to the application of multi-scale community detection in social network research, demonstrating that different resolution levels can reveal distinct patterns of individuals’ selective exposure. This approach is particularly valuable for studying selective exposure, polarization, and echo chambers in multi-party political systems or societies with complex social structures.

## Materials and methods

### Data collection and preprocessing

During the 2022 Brazilian presidential election (from October to November 2022), a national survey was conducted by NetQuest, an international survey company. The data were gathered using NetQuest’s exclusive online panel, using stratified quota sampling aligned with national distributions of age, gender, and geographic area to ensure national demographic representativeness. Statistical tests indicate that the survey participants constitute a representative sample of the national electorate (see Fig B in S1 Text). Participants were enrolled through a double opt-in process and completed the survey online in return for incentives. Approximately 40 questions and 189 variables regarding socio-demographics, news consumption behaviors, and political predispositions were asked in the survey (see the questionnaire in section 2 in S1 Text). We also requested respondents’ consent to provide their Twitter/X handles. Of the 1,018 respondents, 403 consented, and 271 were verified as existing accounts on Twitter/X, accounting for 26.62% of the whole panel.

Based on the 271 survey respondents, we collected the Twitter/X accounts followed by them (57,645 accounts and 73,755 following pairs were collected) using the Twitter/X API. We use “following” relations to represent individuals’ preference to be exposed to information posted by the accounts they follow. We then identify political influencers from the 57,645 accounts. We define political influencers as a composition of both ordinary citizens and celebrities (e.g., politicians, parties, media outlets, journalists, and individual opinion leaders) who satisfy two conditions: 1) with at least more 1,000 followers and 2) indicate political related information in their profiles or are known political figures [47,48] (see section 3 in S1 Text for more details about the identification of political influencers). After filtering out the survey respondents who did not follow any political influencers, we obtained 204 survey respondents (also referred to as political consumers in the text), 2,307 political influencers (also referred to as political suppliers in the text), and 4,107 following pairs between the two groups. Pearson’s chi-squared tests (for categorical variables) and Mann–Whitney U tests (for discrete variables) comparing the demographic distributions of the 204 subsample with the 1,018 survey respondents do not reject the null hypothesis that the 204 samples are drawn from the same distribution as the survey respondents at the 5% significance level (see Fig C in S1 Text). Therefore, the survey respondents who provided their handles are very likely to form a representative sample of the Brazilian electorate.

This study was reviewed and approved by the Ethics Committee of the Faculty of Arts and Social Sciences, University of Zurich. Informed electronic consent was obtained prior to participation via the NetQuest survey platform. Participants were informed of the study purpose, data processing, voluntary participation, and their right to withdraw at any time. Twitter/X data were collected in accordance with the platform’s Terms of Service and API policies and were limited to publicly available content.

### Multi-scale community detection

To analyze the relationships between political consumers and political influencers, we construct a bipartite network 𝒢=(𝒞,𝒮,ℰ), where 𝒞 is the set of ordinary users who primarily consume political information online, and 𝒮 is the set of political influencers who produce such content. The set ℰ contains edges that indicate which users follow which influencers. We then project this bipartite network onto the influencer side, resulting in a new graph ${𝒢}^{𝒮}=(𝒮,{ℰ}^{𝒮})$, where edges in ${ℰ}^{𝒮}$ indicate that the connected influencers are followed by at least one common user. The weight *w*_*ij*_ of an edge between influencers *s*_*i*_ and *s*_*j*_ in the projected graph is equal to the number of shared consumers following both influencers. Similarly, we create a consumer network ${𝒢}^{𝒞}=(𝒞,{ℰ}^{𝒞})$ where edges between two consumer nodes represent the number of common influencers they follow.

We then apply a multi-scale community detection approach to the projected influencer graph ${𝒢}^{𝒮}$. Community detection is the process of identifying groups of nodes that are more densely connected to each other than to the rest of the network. It is a common strategy to study phenomena like selective exposure, polarization, and echo chambers [5,27,59,60]. However, most studies detect communities using single-scale approach, such as modularity optimization [45]. But real-world social networks are usually multi-scale and hierarchical, for example, smaller class groups are embedded within the broader structure of a school [36,37]. Multi-scale community detection approaches allow for the discovery of communities at different resolution scales. This flexibility also enables selecting the scales closest to the real situation among all the experimental scales, depending on the research goals [38].

A variety of techniques have been designed for multi-scale community detection, such as methods with adjustable resolution parameters (e.g., [35,39,40]) and methods which automatically detect relevant scales (e.g., [41–43]). Here, we use Markov Stability [35,44] with automatic scale selection [43] and optimization using the Leiden algorithm [46]. We note that our approach is applicable to the results of any multiscale community detection approach. The rationale of the Markov Stability method is to identify communities as groups of nodes where a random walk remains confined over varying time scales, thus revealing community structures at different resolutions. We give a detailed mathematical explanation of the Markov Stability method in section 5 in S1 Text.

### Multidimensional annotation of identities

We then annotate four identity attributes of political influencer nodes in the network: political ideology, campaign support, social identity, and account type. The data are manually annotated based on the self-presentation of political and social identities in users’ Twitter/X profiles, combined with a formal list of politicians and parties participating in the election. It is shown that users who disclose their political identities publicly are generally more active than those who do not [63]. Almost all political influencers have profile descriptions.

Political ideology is the most traditional and straightforward indicator of individuals’ political opinions and comprises categories {Left, Right, Center}. During the 2022 Brazilian Presidential Election, Lula and Bolsonaro are the two candidates who represent leftist and rightist leaders, respectively. Nevertheless, it is not necessarily the case that all left-wing individuals support Lula and all right-wing individuals support Bolsonaro, due to increasing cleavages on issues and socio-cultural factors, even though there is still significant overlap. To differentiate between these situations, we also annotate them as “Pro-Lula” or “Pro-Bolsonaro” if they support certain candidates during the election campaign.

Many users also indicate their social identities, as cultural issues have become increasingly related to politics. We identify four social identities most frequently revealed in the profiles: Women (in the context of feminism or supporting women’s rights), Religious, Black, and LGBTQ. For religious, Black, and LGBTQ identities, annotation is based on either users’ self-identification with the group or their expression of support for its rights. Note that, unlike the fixed options of political ideology, social identity encompasses more categories; accordingly, we include only those observed in our samples. The same situation applies to account type, which indicates what the accounts represent. We consider media (including media outlets and journalists), politicians, and individual opinion leaders, as they are the most common accounts that produce political information.

As not all political influencers reveal their identities at all dimensions, we assign those unrevealed profiles as “Unlabeled”. The annotation results on political ideology, campaign support, social identity, and account types for influencers in communities of all scale levels are included in section 4 in S1 Text.

### Measurements of selective exposure indices

We introduce five indices – Community Overlap, Identity Diversity, Information Diversity, Structural Integration, and Connectivity Inequality to measure individuals’ selective exposure patterns at different levels. We consider a followship bipartite network between political influencers and survey respondents, and project the network onto both the survey participants’ side and the political influencers’ side. From the projection on the survey participants, we capture the number of influencer communities in which they participate. From the projection on the political influencers, we detect communities and identify the characteristics of influencer communities followed by each survey participant. In this context, we introduce the following measures:

#### Community overlap.

This index is derived from the network’s projection on the survey participants and reflects the number of influencer communities they engage with. It captures how online users are divided into fragmented communities in which individuals primarily interact with others who share similar interests, and how they could act as bridges between multiple communities. This index is based on the phenomenon of cyberbalkanization introduced by Alstyne and Brynjolfsson in their 2005 study [7] which showed that some internet users are more inclined to have diverse interests and engage with multiple communities, while others prefer to remain within a single community. Survey users who connect with multiple communities have a higher potential to act as bridgers—individuals who consume information from a variety of sources [49]. To quantify survey respondents’ capacity to bridge multiple communities, we introduce the Community Overlap Index.

Community Overlap is measured for each consumer node and at each level and corresponds to the number of influencer communities a consumer belongs to. Each consumer node can follow influencers either in a single community or from multiple communities.

At scale *s*, for a consumer node v∈𝒞, let $I(v,E_{i}^{s})$ be an indicator function that is 1 if node *v* belongs to the hyperedge $E_{i}^{s}$- a hyperedge represents at least one connection to influencer community i, with K communities in total, and 0 otherwise. The number of hyperedges node *v* belongs to, i.e., the number of influencer communities that the user follows, is expressed as

$${\text{CO}}^{s}(v)=\sum_{i=1}^{K_{s}}I(v,E_{i}^{s}).$$ (1)

#### Identity diversity.

This index is measured from the network’s projection on the political influencers and indicates the diversity of identities among the influencers engaged by survey participants. According to social identity theory and social categorization theory, people may act based on inter-group behaviors arising from a sense of belonging to certain social groups [50,51]. It can be applied to the political domain, where political identities advance social identities, such as national identities, partisan affiliations, and political ideologies [52]. The diversity of influencers’ political identity in a community is measured by the Identity Diversity Index.

We annotate identities for influencers on four dimensions: political ideology, campaign support, social identity, and account type, as indicated in their profile descriptions. Influencers who strongly exhibit certain attributes may lead individuals to filter into specific community structures. However, not all influencers disclose identities in all dimensions. To address unlabeled accounts, we apply probability assignment, as described below (also see the percentages of unlabeled identities in Figs E–H in S1 Text). We only measure the diversity of labels on the political ideology dimension as it is the most direct indicator of political identity.

We measure this index using the Gini-Simpson Diversity Index, used in fields such as ecology, sociology, and psychology to measure the diversity of types in a dataset [55]. The Gini-Simpson Diversity Index gives the probability that two entities taken at random from the dataset of interest represent different labels. It ranges from 0 to 1, where 0 represents a totally homogeneous distribution of ideology labels, and 1 represents totally diversified labels. For an influencer community $C_{i}^{s}$ at scale *s*, the Gini-Simpson Diversity is computed as

$$\text{IdD}(C_{i}^{s})=1-\sum_{\ell =1}^{L}\left(\frac{n_{\ell }(n_{\ell }-1)}{N(N-1)}\right)$$ (2)

Where *L* is the number of ideological labels (here: “Left”, “Right” and “Center”), $n_{\ell }$ is the number of influencers of label ℓ in community $C_{i}^{s}$, and *N* is the total number of influencers in community $C_{i}^{s}$. As not all the influencers are labeled, we apply two approaches to deal with the missing values: in one approach, we assign probabilities of labels to the unlabeled influencer accounts based on the proportions of labels present in each local community; in the other approach, we only consider the influencer accounts that are labeled. The two approaches yield similar results (see section 6 in S1 Text).

#### Information diversity.

Likewise, this index is computed based on the communities found on the network’s projection on the influencers. Instead of being exposed to certain political identity groups, ordinary users may focus on the information retrieved from specific communities. People may have a preference for certain information sources or opinions due to personal interests or confirmation bias [53,54]. Here, we measure the extent to which individuals are exposed to diverse information using the website domain links shared by political influencers.

Similar to Identity Diversity, we use the Gini-Simpson Diversity Index to measure the diversity of website domains shared by the influencers in that community, which is defined as Information Diversity. The Information Diversity, $\text{InfD}(C_{i}^{s})$ of community $C_{i}^{s}$ at scale *s* is used to measure exposure at the information level and is computed with (2), with *L* as the number of different website domains, $n_{\ell }$ the number of times a website from domain ℓ is shared by an influencer of community $C_{i}^{s}$, and *N* is the total number of links shared by influencers in community $C_{i}^{s}$.

This index measures how diversified the information sources are witnessed by individual consumers who follow that community instead of just the diversity of influencers’ profiles. To achieve better reliability, we remove the communities that share fewer than 100 website domains and have less than 50 percent of influencer sharing links (see section 6 in S1 Text).

#### Structural integration.

This is a structural measure of the influencer communities engaged by survey participants. Each influencer community occupies a distinctive structural position within the network. Some communities are relatively isolated from others, while some are more integrated into the overall structure. In the co-following influencer network, a more isolated community indicates that it caters to a more targeted consumer group, whereas greater integration suggests exposure to a broader audience.

To quantify this, we introduce the Structural Integration Index. This index is derived from the Normalized Cut, a widely used metric in image segmentation and graph partitioning that evaluates the connectivity between two disjoint subsets of nodes in a network [56]. We employ the Normalized Cut to assess the degree of integration of a given community by comparing its internal and external connectivity. For a community $C_{i}^{s}$ at scale *s*, the Structural Integration is defined as:

$$\text{SI}(C_{i}^{s})=\frac{c_{i}^{s}}{2m_{i}^{s}+c_{i}^{s}}+\frac{c_{i}^{s}}{2(m-m_{i}^{s})+c_{i}^{s}}$$ (3)

where $c_{i}^{s}$ is the size of the cut, i.e., the sum of the weights of the edges crossing the boundary of the community $c_{i}^{s}=\sum_{u\in C_{i}^{s},v\notin C_{i}^{s}}w_{uv}$, $m_{i}^{s}$ is the sum of the weights of the edges inside of the community, i.e. $m_{i}^{s}=\sum_{u,v\in C_{i}^{s}}w_{uv}$, and *m* is the sum total of the edge weights in the influencer network, $m=\sum_{u,v\in 𝒮}w_{uv}$. The Structural Integration Index reaches a minimum value of 0 and a maximum value of 2 for two degenerate cuts: the empty cut, and the cut containing all edges. In this work, all the values of the index were between 0 and 1. Lower values indicate stronger isolation with the rest of the network, and larger values reflect greater integration, meaning the community has relatively more or stronger connections to other parts of the network. This measure enables us to evaluate whether survey participants are primarily exposed to influencers from structurally peripheral communities or from those that are more centrally embedded in the network.

#### Connectivity inequality.

This index is also based on the influencer communities but focuses on their internal structures. Some communities exhibit an unequal distribution of (weighted) degrees, with certain nodes having a larger number of connections and potentially playing a more central role, while others display a more even distribution.

We quantify the inequality in the distribution of influencer degree weights—defined as the number of shared survey consumers each influencer has with others within a community—using the Gini Index [57]. It captures the skewness of the degree distribution. A highly skewed distribution may arise due to the presence of “super-influencers”, such as prominent politicians, who share survey consumers with many other influencers. The metric is computed as

$$\text{CI}(C_{i}^{s})=\frac{\sum_{u,v\in C_{i}^{s}}|k_{u}-k_{v}|}{2{N_{i}^{s}}^{2}<k_{i}^{s}>}$$ (4)

where $N_{i}^{s}$ is the number of nodes in the community $C_{i}^{s}$, $k_{u}=\sum_{v\in C_{i}^{s}}w_{uv}$ is the weighted internal degree of node *u* and $<k_{i}^{s}>$ is the average weighted internal degree of the nodes in community $C_{i}^{s}$.

Finally, all influencer community-level indices—Identity Diversity, Information Diversity, Structural Integration, and Connectivity Inequality—are aggregated at the user level for the regression analysis by calculating the weighted average across all influencer communities with which the individual is connected.

Specifically, let ${𝒞}_{u}$ denote the set of influencer communities followed by survey participant *u*, and let $w_{uC_{j}}$ represent the number of influencers in community $C_{j}\in {𝒞}_{u}$ followed by user *u*. For each index I∈{IdD,InfD,SI,CI}, the aggregated individual-level score for user *u* is computed as a weighted average of the community-level values:

$$I_{u}=\frac{\sum_{C_{j}\in {𝒞}_{u}}w_{uC_{j}}\cdot I(C_{j})}{\sum_{C_{j}\in {𝒞}_{u}}w_{uC_{j}}}$$ (5)

This step ensures that communities with more connections to a given user contribute proportionally more to their overall exposure, reflecting the strength of affiliation and potential information influence. In contrast, the Community Overlap Index (${\text{CO}}^{s}(v)$) is inherently computed at the individual level based on the number of influencer communities followed, and thus does not require further aggregation.

### Variable dimension reduction and regression analysis

Having measured the patterns of consumers’ selective exposure, we continue to examine the association of ten groups of individual attributes obtained from the survey: Demographics, News Consumption, Political Communication, Political Identification, Political Engagement, Perceptions of Incivility, Perceptions of Disinformation, Authority Trust, Populism, and Attitudes toward Democracy, with the five selective exposure indices (see section 7 in S1 Text for detailed description of the ten groups of individual attributes).

We perform a regression analysis to explore factors related to the five indices of selective exposure patterns. The independent variables are consumers’ individual attributes. To examine a wide range of consumers’ attributes, we construct 10 groups of 189 variables obtained from the National Survey in Brazil, including Demographics, News Consumption, Political Communication, Political Identification, Political Engagement, Perceptions of Incivility, Perceptions of Disinformation, Authority Trust, Populism, and Attitudes to Democracy. These groups encompass individuals’ social, political, and media attributes. We then reduce the dimensions of variables in each group by applying PCA, retaining only one variable for each significant dimension, resulting in 25 variables in total. (See section 7 in S1 Text).

The dependent variables are measurements of five indices of selective exposure patterns. As mentioned before, except for Community Overlap, the other four indices—Identity Diversity, Information Diversity, Structural Integration, and Connectivity Inequality—are initially measured based on influencer communities. We transform these four indices onto individual levels by calculating the average values of influencer communities each consumer interacts with, weighted by the number of links between consumers and influencers in each community.

Finally, we conduct a regression analysis between the reduced attribute variables and the transformed measurements of five indices. We employ a forward selection approach with BIC to select the appropriate regression models for all the selective exposure indices; variables are added iteratively until the BIC does not improve anymore. We use a Zero-truncated Negative Binomial regression (implemented with *vglm* R package, family = *posnegbinomial*) for Community Overlap, since it accepts values in positive integers, and Beta regression with a logit link function for the other indices, which all range between zero and one (implemented with *betareg* R package [58]).

For a more intuitive understanding of our methodological design, please refer to Fig A in S1 Text.

## Results

### Multidimensional profiles of political influencers and their interconnections

We build a bipartite network between 204 political consumers and 2,307 political influencers by combining datasets of surveys and Twitter/X. Political consumers are sampled from respondents of a national survey conducted during the 2022 Brazilian Presidential Election. Political influencers are identified based on the Twitter/X accounts followed by these survey respondents and can be politicians, media, and individual opinion leaders. Finally, 4,107 following pairs are found in the followship network.

**Fig 1:**
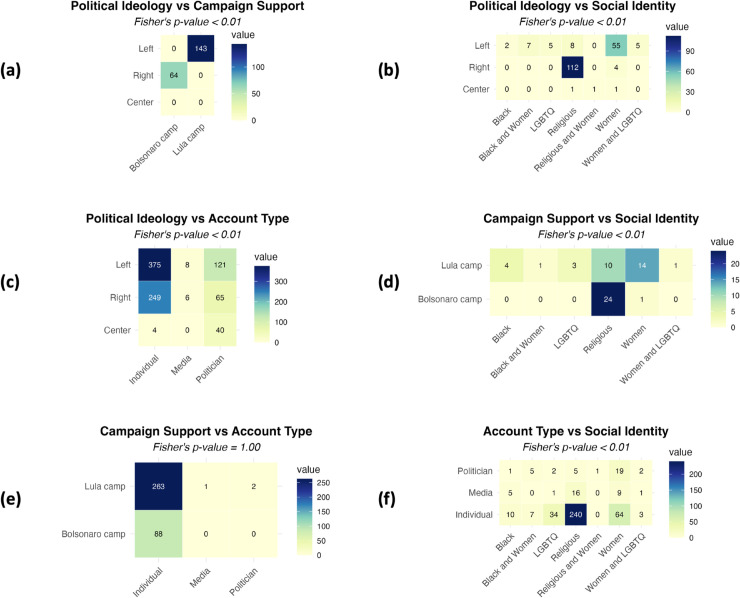
Contingency tables of multidimensional annotations of political influencer Twitter/X accounts. Influencer accounts are annotated based on the dimensions of Political Ideology (*N* = 886), Campaign Support (*N* = 356), Social Identity (*N* = 438), and Account Type (*N* = 2,129). Correlations among categories are examined for each dimension pair (a–f). A multivariate Fisher’s exact test is applied to each contingency table to assess dependencies between dimensions, with significance levels indicated. In Social Identity, *Women* denotes feminism or support for women’s rights; *Religious*, *Black*, and *LGBTQ* refer to self-identification or expressed support for the rights of these groups.

Based on our annotation of political influencers on four dimensions - political ideology, campaign support, social identity, and account type, We find that influencers’ attributes across different dimensions are correlated to some degree (see Fig 1). A Fisher’s exact test confirms that accounts with left-wing ideologies are associated with the support of Lula - the leftist leader and current president of Brazil and advocate for women’s rights (Fisher’s Exact Test *p*-value <0.01). Conversely, right-wing accounts are more likely to support Bolsonaro - the rightist leader, former president of Brazil, and be associated with religious groups (*p* < 0.01). Most accounts that express support for electoral candidates are from individual opinion leaders, showing only weak dependency between dimensions of Campaign Support and Account Type (*p* = 1.00). However, left-wing accounts demonstrate higher proportions of politician accounts compared to others, and religious accounts show higher correlations with individual opinion leaders compared to other identities (*p* < 0.01).

These correlation patterns can suggest a dependency relation or potentially hierarchical relation between dimensions. To understand whether there is a more complex structure of individuals’ selective exposure to political influencers, we investigate how the political influencers are organized in the network with a multi-level analysis.

### Hierarchical structure of online political selective exposure

**Fig 2:**
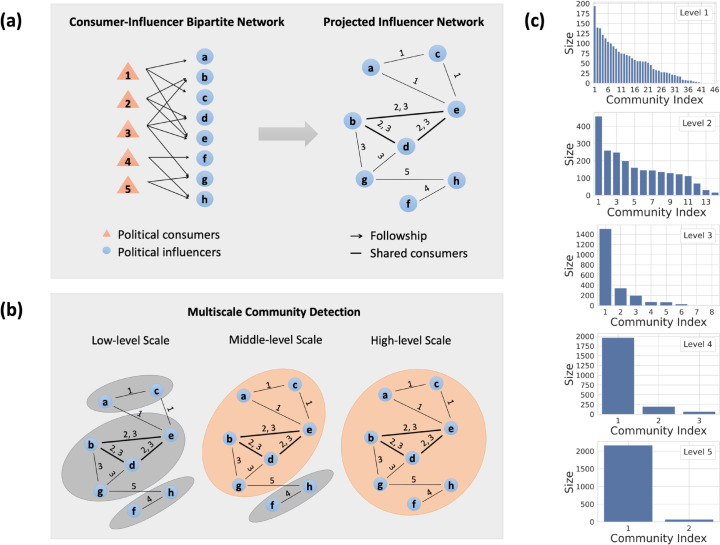
Schematic diagram of network construction and community detection. (a) The procedure used to construct the bipartite network between political consumer nodes (orange triangles) and political influencers nodes (blue circles). We project the bipartite network onto political influencers. The directed arrows between consumers and influencers represent the following relationships on Twitter/X. The undirected lines between influencers indicate their shared consumers, weighted by the number of shared consumers. (b) Illustration of multi-scale community detection in the projected influencer network. At the lower level, there are more communities, and the size of each community is smaller (shown in grey). At the higher level, the smaller communities merge, resulting in fewer, larger communities (shown in orange). (c) Histograms of community sizes at the five detected levels.

We then project the bipartite network onto the influencer side and conduct multi-scale community detection on the influencer-projected network to measure the complexity of individuals’ selective exposure structures (see the schematic diagram in Fig 2(a) and 2(b) and the Materials and methods section for more details).

To capture the multi-scale and hierarchical organization of real-world social networks [36,37], we conduct multi-scale community detection [35,43] on the influencer network (see details in the Materials and methods section and section 5 in S1 Text). We detect five significant levels (or scales). The number of influencer communities detected at five levels is shown in Fig 2(c), ranging from 46 communities at the lowest level and 2 communities at the highest level. In this setting, a community represents a set of political influencers more likely to be co-followed by the same set of consumers.

**Fig 3:**
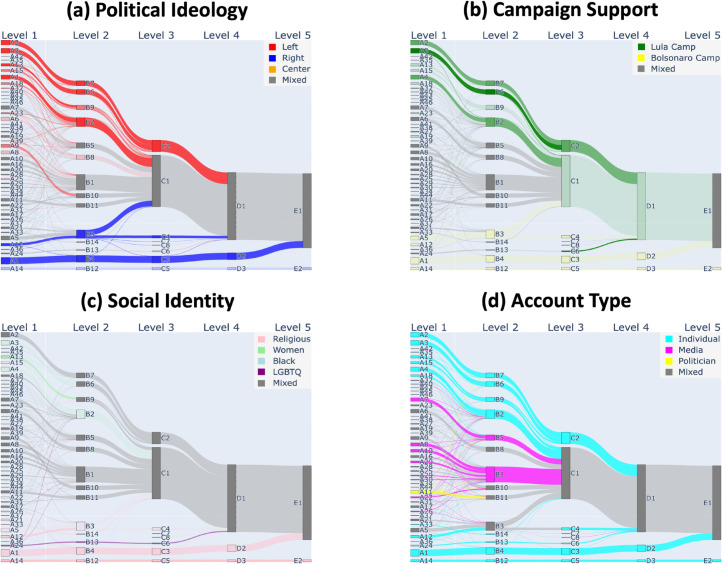
Alluvial diagram showing the hierarchical organization of political influencer communities across five levels. The different panels display the labeling of political influencer communities according to the following dimensions: Political Ideology (a), Campaign Support (b), Social Identity (c), and Account Type (d). The partition of the influencers in communities from a granular level to coarser levels is shown by the different grouping and arrangement of the bundles. We assign a color to a community if more than half of the influencer accounts within the community belong to one category. The transparency of the colors is proportional to the homogeneity of the labels; the higher the proportion of the majority category, the more salient the color. We only color communities when more than 30% of influencer accounts are annotated. In Social Identity, *Women* denotes feminism or support for women’s rights; *Religious*, *Black*, and *LGBTQ* refer to self-identification or expressed support for the rights of these groups.

Fig 3 shows the results of the multi-scale community detection of the influencer network as an alluvial diagram, revealing its hierarchical structure. As we can see, at the most granular level (Level 1), diverse communities are formed based not only on ideologies but also on political support, social group affiliation, and account types. At the second scale (Level 2), communities with mostly the same categories merge, resulting in more unified left-wing and right-wing communities, as well as distinct Lula and Bolsonaro camps. From level 3 onward, the left-wing and right-wing communities, along with Lula and Bolsonaro supporters, merge together. At the same time, many categories based on social identity and account types become less prominent. By levels 4 and 5, we observe a large mixed community and predominantly right-wing communities and Bolsonaro supporters on the side, who are more isolated and mainly come from religious groups and individual opinion leaders.

The results show that a single scale in community detection may not always detect the polarized selective exposure pattern between left-wing and right-wing groups. Lower-level scales can uncover more fragmented communities nested within ideological groups, while higher-level scales may capture more integrated ideological communities. Our findings also suggest that the higher-level ideological segmentation in Brazil is asymmetric, with some right-leaning communities being more isolated while others are integrated into larger, more mixed communities. This aligns with findings on the asymmetric political news consumption in the United States [6].

### Measurements of online political selective exposure patterns

**Fig 4:**
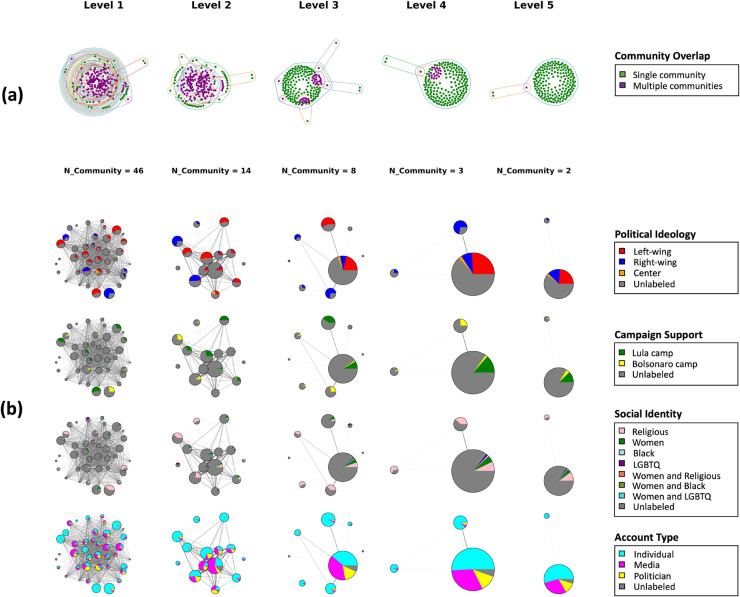
Projection of the consumer-influencer bipartite network onto both the consumer and influencer sides. (a) Influencer communities projected on the consumer network. Each node represents a consumer who can follow single or multiple influencer communities. Consumers following the same communities are encircled. Consumer nodes that follow a single community are annotated in green, while those that follow more than one community are annotated in purple. All five levels are displayed. (b) Projected pie-chart graph of the influencer community network. The nodes are influencer communities, indicated by a pie chart of labels on four dimensions: Political Ideology, Campaign Support, Social Identity, and Account Type. Proportions of categories in each dimension are shown inside the pie chart. Edges between two nodes denote the consumers who follow both influencer communities. In Social Identity, *Women* denotes feminism or support for women’s rights; *Religious*, *Black*, and *LGBTQ* refer to self-identification or expressed support for the rights of these groups.

We also project the consumer-influencer bipartite network onto the consumer side. We group consumers together if they follow political influencers in the same influencer community. The consumers can, therefore, be involved in multiple or just a single influencer community (see Fig 4(a)). We observe that as the level increases, the number of individuals involved in multiple influencer communities decreases. In contrast, the number of individuals who belong to only a single influencer community increases.

Consumers who are involved in multiple influencer communities create closer connections between these influencer communities. We visualize the influencer community network connected by shared consumers in Fig 4(b), where each pie chart represents an influencer community node and shows the proportions of different identity categories across four dimensions. The edges represent consumers who follow both communities. At Level 1 and 2, we observe that influencer communities closely connected by shared consumers exhibit high proportions of left-wings and media categories. Consumers following these categories of influencer accounts are more versatile and fragmented than others, and this pattern can be mainly observed at the lower level.

**Fig 5:**
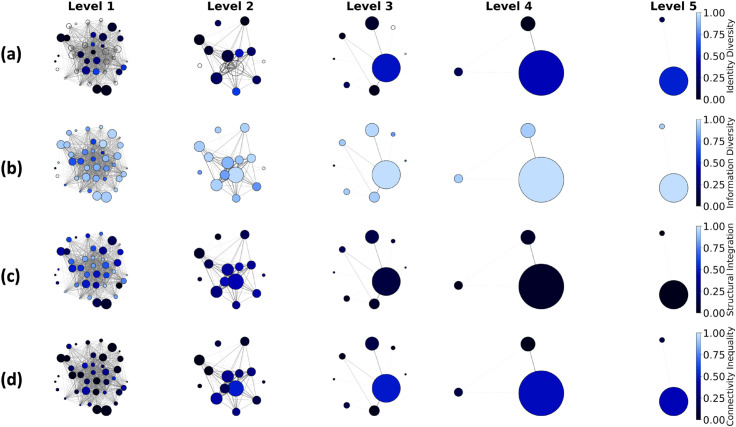
Visualization of measurements of four community-level indices in the influencer community network. (a) Identity Diversity, (b) Information Diversity, (c) Structural Integration, and (d) Connectivity Inequality. The nodes in each network represent detected influencer communities, and the edges represent the shared consumers between two influencer communities. The area of each node is proportional to the number of political influencers in the community. The color spectrum ranges from black to blue, with a darker color indicating more homogeneous ideological identities and domain information, more integration from other communities, and a more equal distribution of degree weights.

We also show the values of the four community-level indices in Fig 5. The granular levels (Level 1 and Level 2) demonstrate diverse values across the four indices. As the levels increase, there is a large merged influencer community where the political ideologies are more diverse and the (weighted) degree distribution is more unequal compared to other communities. Information Diversity and Structural Integration show little distinction among communities at higher levels.

### Individual attributes related to multi-level selective exposure patterns

We continue to investigate how certain consumer attributes are related to selective exposure patterns at various levels with a regression analysis. Individual attributes are conceptualized into 10 groups, encompassing 189 variables, including Demographics, News Consumption, Political Communication, Political Identification, Political Engagement, Perceptions of Incivility, Perceptions of Disinformation, Authority Trust, Populism, and Attitudes toward Democracy. We conduct Principal Component Analysis (PCA) within each variable group to address the issue of multicollinearity. This results in 25 variables after PCA dimension reduction, which are then regressed against the five indices. It is worth noting that we convert the four community-level indices—Identity Diversity, Information Diversity, Structural Integration, and Connectivity Inequality—into individual-level indices by calculating the average values of the influencer communities each consumer interacts with, weighted by the number of influencers each consumer follows (see mathematical definitions in the Materials and methods section).

We identify significant explanatory variables among the 25 initial variables for each index at each level using a forward selection approach based on the Bayesian Information Criterion (BIC). This model selection procedure aims to find the statistical model with the fewest explanatory variables by iteratively adding them until the statistical significance of the model, as measured by the BIC, no longer improves (see section 7 in S1 Text for more details).

**Fig 6:**
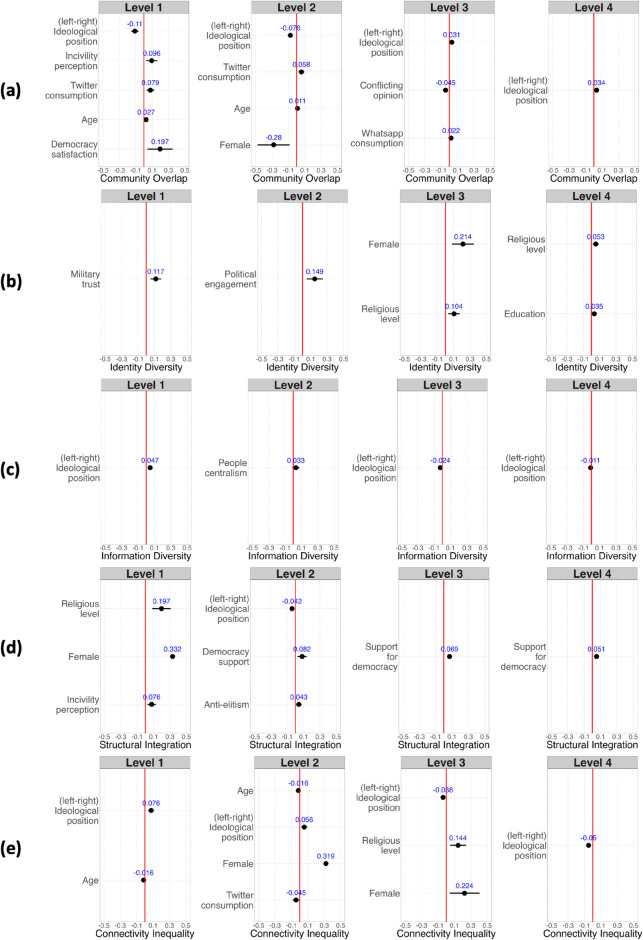
Regression plot of significant individual attributes related to selective exposure patterns. Regression analyses of consumers’ attributes on Demographics, News Consumption, Political Communication, Political Identification, Political Engagement, Perceptions of Incivility, Perceptions of Disinformation, Authority Trust, Populism, and Attitudes toward Democracy are conducted across five indices of selective exposure patterns: (a) Community Overlap, (b) Identity Diversity, (c) Information Diversity, (d) Structural Integration, and (e) Connectivity Inequality at four network community levels. The variables displayed are significant factors resulting from a forward selection based on BIC. The coefficient values are displayed in blue, and the confidence intervals at the 95% confidence level are shown with error bars. The red lines indicate the 0 value.

We fit the minimal explanatory variables into the regression model for each index and each level. After conducting distribution checks for the values of dependent variables, the zero-truncated Negative Binomial Regression model is chosen as the basic model for the Community Overlap Index. For the indices of Identity Diversity, Information Diversity, Structural Integration, and Connectivity Inequality, the Beta Regression model is employed. For more details, refer to the Materials and methods section and to section 7 in S1 Text. The regression results for the first four levels are shown in Fig 6.

For the Community Overlap Index, we can observe that the ideological position (measured on a scale from 0 to 10, where 0 means “Very left-wing” and 10 means “Very right-wing”) of individuals plays a significant role in explaining their involvement in multiple communities at all levels. However, it is not the only factor; at lower levels of communities, more factors contribute to the versatile engagement of consumers in influencer communities (see Fig 6(a)). The impact of ideological position corresponds to the fragmentation pattern displayed in Fig 4(b), suggesting that left-wing influencer communities are more likely to be shared by consumers with high bridging capacity. Another two significant factors that remain at the first two levels are the frequency of news consumption on Twitter/X and age. At Level 3, the sign of the ideological position coefficient changes, demonstrating a turning point from which right-wing consumers are more inclined to be involved in multiple influencer communities. This is because as the left-wing influencers merge, most of the remaining isolated communities are right-wings. The ideological position plays an increasingly important role at higher levels, especially at Level 4, where only the ideological position remains significant.

The other community-level indices show correlations with different attributes, but similarly to the Community Overlap, they demonstrate various associations across levels. For instance, military trust, with collinearity among variables such as trust in the police and trust in the government, and the frequency of participating political activities primarily explain Identity Diversity at Level 1 and Level 2 (see Fig 6(b)). However, demographic variables play a more important role at Levels 3 and 4, where the religious level is consistent at both levels.

The ideological position of individuals plays a more significant role in Information Diversity compared to other indices. As shown in Fig 6(c), the ideological position is the only significant factor at Levels 1, 3, and 4, and a sub-dimension of populist ideology—people centralism, which is the demand for prioritizing the rights of the majority, is a significant factor at Level 2.

For Structural Integration, there are many explanatory factors at the finer levels, while the degree of integration of communities is mainly related to consumers’ support for democracy at Levels 3 and 4 (see Fig 6(d)). Regarding Connectivity Inequality, similar to Community Overlap, at lower scales, ideological position, as well as a variety of other factors such as age, gender, religious level, and the frequency of news consumption on Twitter/X, have explanatory power. Only the ideological position remains significant at Level 4 (see Fig 6(e)).

## Discussion

The findings of this study reveal the intricate and hierarchical nature of individuals’ political selective exposure in the Brazilian Twitter/X space. By employing a multidimensional annotation design, we discover that attributes of political influencers are correlated across different dimensions. For instance, left-wing accounts are correlated with attributes of supporting Lula and advocating for women’s rights. In contrast, right-wing accounts are more likely to be associated with Bolsonaro supporters and religious believers. This highlights political influencers involved in homogeneous left-wing and right-wing communities or Lula-camp and Bolsonaro-camp also resemble each other on demographic grounds, even though it does not apply for all the communities.

In the long history of political studies, demographics such as gender, race, ethnicity, and religion have been related to ideological positions and voting behaviors [64]. In recent years, demographic cleavages have become more significant due to the emergence of far-right and populist political leaders in political campaigns [65]. Layton et al. (2021) demonstrated a pattern of new alignments in Brazil’s electorate since the 2018 presidential election, showing that political groups are now split more based on demographics and issues rather than purely partisanship [66]. In particular, the far-right candidate Bolsonaro has attracted new supporters by creating demographic polarization beyond the traditional left-versus-right division. For instance, his misogynistic views on gender, racially offensive remarks to Black activists, and evangelical propositions have played a role in this polarization [66]. The strong correlations between political ideology and other dimensions suggest that a new trend of political division might be ongoing.

In light of this background, media scholars and political scientists have gradually shifted from the traditional focus on selective exposure, polarization, and echo chambers of political ideology and partisanship to a wider range of divisions such as issues, demographics, and beliefs [67]. However, effective methods to detect such selective exposure at various levels are still lacking. This study provides a novel perspective by utilizing a multi-scale community detection approach to identify different patterns of selective exposure. This approach can also be applied to research on similar topics, such as polarization and echo chambers.

The hierarchical patterns observed through multi-scale community detection demonstrate the importance of levels in understanding the complexity of political selective exposure (RQ1). As community detection moves to coarser levels, the communities in which survey participants are involved change accordingly. These changes can be captured by various measurements, including Community Overlap, Identity Diversity, Information Diversity, Structural Integration, and Connectivity Inequality. For instance, participants may shift from engaging with multiple communities to engaging with only one as the level becomes more aggregated (Community Overlap), and the communities they engage with may become more homogeneous or more diverse in terms of identity (Identity Diversity). These measurements offer a quantitative perspective for understanding how selective exposure happens at various resolution levels. At the most granular level, diverse communities form based on various factors, including political ideology, candidate support, social group affiliation, and account type. As the scale increases, these communities gradually merge, forming more unified—albeit asymmetric—ideological groups. This process continues until higher scales predominantly reveal right-wing communities and Bolsonaro supporters as homogeneous and isolated communities, which are mainly associated with religious groups and individual opinion leaders.

We find that Community Overlap is a useful measure of a consumer’s selective exposure patterns. Many variables, such as demographics, news consumption frequency, incivility perception, and ideological position, are found to be related to individuals’ versatility in connecting to influencer communities. However, only ideological position is significant at the coarsest level. Our investigation into the related factors of selective exposure patterns reveals that different factors play a role in multi-level selective exposure (RQ2). We reveal a more nuanced picture of political information filtering than traditional left–right ideological models alone can capture. Specifically, the emergence of fine-grained communities nested within broader ideological camps suggests that demographic attributes (e.g., age, gender, religion), social media experience (e.g., Twitter/X consumption frequency, incivility perception), as well as other factors, may exert greater influence at localized scales. For example, we find that the gender female emerges as a significant factor at the second level, associated with engagement in fewer communities. Indeed, Fig 3(c) shows that most influencers advocating for women’s rights form a single, homogeneous community at this level, implying that individuals’ exposure to political content is not only shaped by ideological alignment but also by socio-demographic proximity and information consumption habits, which may reinforce specific opinions or beliefs within subgroups. For instance, the alignment of social and political identities might lead to increased polarization and group-based animosity, potentially resulting in toxicity and severe attacks. This multilevel approach thus offers a powerful lens for understanding how political attitudes are shaped in complex media ecosystems, where micro-level affiliations and macro-level ideologies intersect. Moreover, this framework has broader applicability beyond the Brazilian context, offering a valuable tool for studying political communication dynamics in other multi-party democracies or conducting cross-system comparison. By uncovering the layered mechanisms underlying selective exposure, our approach advances understanding of the complex drivers of fragmentation and mitigates the risk of overgeneralizing conclusions to entire ideological groups. Methodologically, it also suggests that online communities detected at a single level might miss important information; therefore, various levels should be examined in relevant studies.

This study builds both theoretical and methodological foundations for exploring multilevel selective exposure and related concepts such as polarization, and echo chambers. Although the survey sample size is limited in this study, this does not influence the validity of the insights brought by our novel multi-scale approach, which would also apply to larger samples. Additionally, we do not screen for potential Twitter/X bots. However, as influencers who are potentially bots, or followed by many bots, have been willingly followed by survey users and participate in their information exposure, including them is necessary to correctly capture the selective exposure of survey users. We also only use following relations rather than other types of interactions. This is because we focus on information exposure rather than engagement. It is worth noting that recommendation algorithms can also influence information exposure independently of whom the survey participants follow. Future research could explore other forms of interaction and assess the role of recommendation algorithms to complement this approach. Methodologically, when projecting the bipartite network, this work retains all link information in the projection. We note that alternative projection methods for bipartite networks, such as the fixed degree sequence model or the stochastic degree sequence model, that extract a backbone preserving only the most significant links [68] could be used in future work. Additionally, for research aimed at uncovering nested structures and fine-grained subgroups within political systems—where actors may belong to different communities at different scales, as in this study—the Markov stability multiscale approach offers distinct advantages over modularity-based methods. However, alternative approaches may be more suitable depending on the research goals. For instance, overlapping community detection methods are well suited for capturing multifaceted affiliations, where nodes can simultaneously belong to multiple communities at the same scale. If the main goal is to reveal both hierarchical structures and multidimensional affiliations of users at each scale, alternative methodologies such as multi-scale overlapping community detection can be explored. In summary, this study demonstrates the necessity of employing multi-level approaches to effectively capture the complexity of political selective exposure in countries with multi-party systems and complex social structures. Our results highlight that political communities are not monolithic; rather, they are characterized by intricate interconnections across various dimensions and scales. Single-scale analyses may overlook critical aspects of community formation and evolution, potentially leading to incomplete or biased understandings of online political patterns. Future research should continue to refine such multi-scale methodology, potentially combining with content and issues, to interpret the multi-level behaviors of minority groups and radical ideological groups.

## Supporting information

S1 Text Survey questionnaire, additional methods, and supplementary results. (PDF)
