# Supplementary material for: A multilevel network approach to revealing patterns of online political selective exposure
Source: PLoS One. 2025 Sep 22;20(9):e0332663. doi: 10.1371/journal.pone.0332663 (PMC12453245; doi:10.1371/journal.pone.0332663)
Supplement: S1 Text — (PDF) [file pone.0332663.s001.pdf]

Supplementary Information for "A multilevel network approach to revealing patterns of online political selective exposure"

Yuan Zhang[1,*], Laia Castro[2], Frank Esser[1] and Alexandre Bovet[3,4,*]

[1]Department of Communication and Media Research, University of Zurich, Switzerland
[2]Department of Political Science, University of Barcelona, Spain
[3]Department of Mathematical Modeling and Machine Learning, University of Zurich, Switzerland
[4]Digital Society Initiative, University of Zurich, Switzerland.
* y.zhang@ikmz.uzh.ch, alexandre.bovet@uzh.ch

1. Overview of Methodological Design

The following flowchart illustrates the overall methodological design of this study (see Fig A).

2. Survey Questionnaire

Section I. Demographics

(Age) How old are you?

(Gender) Are you...?

1. Male
2. Female
3. Other

(Ethnic) How do you describe yourself? (select all that apply)

1. White
2. Black
3. Mixed
4. Asia
5. Indigenous
6. Other (*write in*): ________________________
7. Prefer not to answer

(PT version) **(Etnia)** A sua cor ou raça é? (RU) Nesta pergunta é possível assinalar somente uma alternativa.

1. Branca

2. Preta
3. Parda
4. Amarela
5. Indígena
6. Outro (*anote*): ________________________
7. Prefiro não responder

(Education) What is the highest degree or level of school you have completed? (If you're currently enrolled in school, please indicate the highest degree you have received.)

1. Até a Pré Escola
2. Até a 4ª série/ 5º ano do Ensino Fundamental
3. Até a 8ª série/ 9º ano do Ensino Fundamental
4. Até o 1º ano do Ensino Médio
5. Até o 2º ano do Ensino Médio
6. Até o 3º ano do Ensino Médio
7. Superior Incompleto
8. Superior Completo
9. Pós-graduação ou Mestrado
10. Doutorado

(PT version) **(Educação)** Até que ano da escola você cursou? (RU) Nesta pergunta é possível assinalar somente uma alternativa.

1. Até a Pré Escola
2. Até a 4ª série/ 5º ano do Ensino Fundamental
3. Até a 8ª série/ 9º ano do Ensino Fundamental
4. Até o 1º ano do Ensino Médio
5. Até o 2º ano do Ensino Médio
6. Até o 3º ano do Ensino Médio
7. Superior Incompleto
8. Superior Completo
9. Pós-graduação ou Mestrado
10. Doutorado

(Religion) What is your present religion, if any?

1. Roman Catholic
2. Protestant evangelical or pentecostal
3. Protestant non-evangelical
4. Non-christian Oriental Religions (Islamism, Hinduism, Buddhism)
5. Jeova's Witnesss
6. Afro-brazilian religions (Umbanda, Candomble)
7. Kardecist
8. Jewish
9. Other religions
10. Is religious but doesn't follow any religion / Agnostic
11. Atheist
12. Prefer not to answer

(PT version) **(Religião)** Qual a sua religião ou culto? (RU) Nesta pergunta é possível assinalar somente uma alternativa.

1. Católica Apostólica Romana
2. Protestante evangélico e pentecostal (Igreja Universal, Quadrangular, Batista, Adventista, etc.)
3. Protestante não evangélico (Calvinista, Luterano, Metodista, Anglicano, etc)
4. Religiões orientais não cristãs (Islamismo, Budismo, Hinduísmo)
5. Testemunha de Jeová
6. Religiões afro-brasileiras (Candomblé, Umbanda)
7. Kardecista, espírita
8. Judeu
9. Outras religiões
10. É religioso mas não segue nenhuma / Agnóstico
11. Ateu
12. Prefiro não responder

(Religious Level) Regardless of whether you belong to a particular religion, how religious would you say you are?

1. Very religious
2. Moderately religious
3. Somewhat religious

4. Not at all religious
5. Prefer not to answer

(Living) Which description best describes the area where you live?

1. A big city
2. The suburbs or outskirts of a big city
3. A town or a small city
4. A country village
5. A farm or a home on the countryside
6. Don't know

(Income) Which of the following income brackets applies to your household income, including you and everyone who lives with you? Please include all sources of income, such as salaries, pensions etc.

1. Up to R$ 1.212,00
2. R$ 1.212,01 to R$ 2.424,00
3. R$ 2.242,01 to R$ 3.636,00
4. R$ 3.636,01 to R$ 6.060,00
5. R$ 6.060,01 to R$ 12.120,00
6. R$ 12.120,01 to R$ 24.240,00
7. R$ 24.240,01 to R$ 36.360,00
8. More than R$ 36.360,00
9. I don't know / Prefer

(PT version) **(Renda)** Em qual das faixas abaixo estava a renda total da sua família no mês passado, somando as rendas de todas as pessoas que moram com você, inclusive a sua? Nesta pergunta é possível assinalar somente uma alternativa.

1. Até R$ 1.212,00
2. De R$ 1.212,01 até R$ 2.424,00
3. De R$ 2.242,01 até R$ 3.636,00
4. De R$ 3.636,01 até R$ 6.060,00
5. De R$ 6.060,01 até R$ 12.120,00
6. De R$ 12.120,01 até R$ 24.240,00
7. De R$ 24.240,01 até R$ 36.360,00
8. Mais de R$ 36.360,00
9. Não sei/ Prefiro não responder

Section II. News Consumption

(News) In general, how often would you say you read or watch news and get information on the following platforms?

a. Television news

(- Never, Less often than once a month, Once a month, Once every 2 to 3 weeks, 1-2 days a week, 3-4 days a week, 5-6 days a week, Once a day, More often than once a day +)

b. National newspapers

(- Never, Less often than once a month, Once a month, Once every 2 to 3 weeks, 1-2 days a week, 3-4 days a week, 5-6 days a week, Once a day, More often than once a day +)

c. Regional newspapers

(- Never, Less often than once a month, Once a month, Once every 2 to 3 weeks, 1-2 days a week, 3-4 days a week, 5-6 days a week, Once a day, More often than once a day +)

d. Radio news

(- Never, Less often than once a month, Once a month, Once every 2 to 3 weeks, 1-2 days a week, 3-4 days a week, 5-6 days a week, Once a day, More often than once a day +)

e. Online news sources (e.g., online newspaper, online magazines, etc.)

(- Never, Less often than once a month, Once a month, Once every 2 to 3 weeks, 1-2 days a week, 3-4 days a week, 5-6 days a week, Once a day, More often than once a day +)

f. News via social media

(- Never, Less often than once a month, Once a month, Once every 2 to 3 weeks, 1-2 days a week, 3-4 days a week, 5-6 days a week, Once a day, More often than once a day +)

(News Social Media) In a typical week, which of the following sites or mobile apps do you use to find, read, watch, share, or comment news? (Please, select all that apply)

1. WhatsApp
2. YouTube
3. Telegram
4. Twitter
5. Facebook
6. Other (please specify)

(Campaign News) Now let's talk about your political news habits to gain knowledge about the Presidential election campaign. How often would you say you read or watched political and campaign news and information on the following platforms in the past week? (0 days - 7 days)

a. Television news

b. National newspapers

c. Regional newspapers

d. Radio news

e. Online news sources (e.g., online newspaper, online magazines, etc.)

f. News via social media

(Social Campaign Information Engagement) How often would you say you read or watched political information & campaign information on the following social media platforms over the past week? (0 days - 7 days - Don't know)

(Social Campaign Information Sharing) How often would you say you shared or liked (on Twitter: also retweet) political information & campaign information on the following social media platforms over the past week? (0 days - 7 days - Don't know)

(Social Campaign Information Commenting) How often would you say you commented or posted (on Twitter: tweet or reply) political information & campaign information on the following social media platforms over the past week? (0 days - 7 days - Don't know)

Platforms:

1. Twitter
2. YouTube
3. Facebook
4. Whatsapp
5. Telegram
6. Other (please specify)

(Election Information Received on WhatsApp/Telegram) Have you received information about the election in your WhatsApp or Telegram groups from:

1. Family and friends
2. People I don't know very well (e.g., colleagues, acquaintances, neighbors)
3. People I don't know personally
4. I have not received information about the election in my WhatsApp or Telegram groups (97)

(Election Discussion Participation on WhatsApp/Telegram) Have you participated in a discussion about the elections in your WhatsApp or Telegram groups with:

1. Family and friends
2. People I don't know very well (e.g., colleagues, acquaintances, neighbors)
3. People I don't know personally

4. I have not participated in a discussion about the election in my WhatsApp or Telegram groups (97)

(Public Political Information Group) Do you belong to any public group that shares information about politics and/or the Presidential election (i.e., public groups are accounts anyone can join by using a URL link)?

1. Yes
2. No

Section III. Political Communication

(Online Political Discussion Fatigue) Thinking about the posts you see on social media about politics, to what extent do you agree or disagree with the following statements? Strongly disagree (1) - Strongly agree (7)

a. I like seeing lots of posts and political discussions on social media.

b. I am worn-out by how many political posts and discussions I see on social media.

c. I don't feel strongly about these discussions one way or another.

Conflict Orientation) Please indicate your level of agreement with the following statements. Strongly disagree (1) - Strongly agree (7)

a. I enjoy challenging the opinions of others.

b. I find conflicts exciting.

c. I hate arguments.

d. I feel upset after an argument.

e. Arguments don't bother me.

(Political Discussion Frequency) In general, how often do you discuss politics with...

a. your family and/or friends?

b. colleagues, acquaintances, and/or neighbors?

c. people online whom I know well (e.g., on social media)?

d. people online whom I don't know well or I don't know personally (e.g., on social media)?

(Encounter of Diverse Opinions) When you talk to people in your surrounding about the 2022 Brazilian Presidential Election, how often do you encounter opinions that are NOT in line with your own opinion? Strongly disagree (1) - Strongly agree (7)

a. your family and/or friends

b. colleagues, acquaintances, and/or neighbors

c. People online whom I know well (e.g., on social media)?

d. People online whom I don't know well or I don't know personally (e.g., on social media)?

Section IV. Political Identification

(Party Affiliation) Do you consider yourself to be close to any particular political party? If so, which party do you feel close to?

1. MDB
2. PT
3. PDT
4. Novo
5. PL
6. PCB
7. PSTU
8. Democracia Cristã
9. Unidade Popular
10. Pros
11. PTB
12. União Brasil
13. Other party (please specify): ____________________
14. No, I do not feel close to any particular party.

(Degree of Closeness to Party) How close do you feel to this party?

(- Not very close ... Very close +)

(Political Position) In political matters people talk of "the left" and "the right". What is your position? (Please indicate your views using any number on a scale from 0 to 10, where 0 means "Very left-wing" and 10 means "Very right-wing")

(0 Very left-wing ... Very right-wing 10)

(Candidate Likeability) To what extent do you like or dislike each of the following party candidates? (Please use the scale below, where 0 is 'strongly dislike' and 10 is 'strongly like'. Rate just the leaders that you know)

(0 Strongly dislike ... Strongly like 10)

Candidates:

a. Simone Tebet (MDB)
b. Lula (PT)
c. Ciro Gomes (PDT)

d. Felipe D'Ávila (Novo)

e. Jair Bolsonaro (PL)

f. Sofia Manzano (PCB)

g. Vera Lúcia Salgado (PSTU)

h. Constituinte Eymael (Democracia Cristã)

i. Léo Péricles (Unidade Popular)

j. Pablo Marçal (Pros)

k. Padre Kelmon (PTB)

l. Soraya Thronicke (União Brasil)

(Party Likeability) To what extent do you like or dislike each of the following parties? (Please use the scale below, where 0 is 'strongly dislike' and 10 is 'strongly like'. Rate just the parties that you know)

(0 Strongly dislike ... Strongly like 10)

Parties:

a. MDB

b. PT

c. PDT

d. Novo

e. PL

f. PCB

g. PSTU

h. Democracia Cristã

i. Unidade Popular

j. Pros

k. PTB

l. União Brasil

Section V. Political Engagement

(Civic and Political Engagement) During the past 12 months, how often have you done any of the following (Never, rarely, from time to time, frequently, very often):
Institutional/Electoral campaign

a) Contacted an elected official (by letter, telephone or email)

b) Donated money to a political party, a political organization, or a candidate running for public office

c) Volunteered for a political party or campaign (like distributing leaflets)

d) Participated in a political meeting

e) Discussed social and political issues with others

f) Encouraged others to take action about political issues

g) Encouraged others to vote

Protest

a) Signed a petition

b) Participated in a march or street demonstration

c) Refused to buy, or boycotted, certain products or services because of the social or political values of the company

d) Joined unofficial strikes

Civic engagement

a) Volunteered for a non-profit organization or charity (like environmental organization or Red Cross)

b) Donated money to a non-profit or charity organization (like environmental organization or Red Cross)

Online participation

a) Posted your own political opinion on social media

b) Commented on a political post on social media

c) Shared a political post on social media

d) Followed a political party, a candidate or a politician on social media

e) Changed your profile picture on social media to support a social cause or in response to a current event

(Interest in Politics) Generally speaking, how interested are you in politics?

(- Not at all interested ... Very interested +)

(Following Brazilian Politicians or Political Parties) Do you regularly follow any Brazilian politician or political party on...
Rows

1. No, I don't follow any politicians or parties

2. Yes, I follow 1 politician or party.

3. Yes, I follow some politicians or parties.

4. Yes, I follow many politicians or parties.

Platforms

a Facebook

b Twitter

c Other social media (please specify)

(Type of Politicians or Political Parties Followed) What type of politicians or political parties do you follow?

1. Those whom I share similar views
2. I follow political figures and parties with a diversity of views
3. I do not share their views

Section VI. Incivility Perception

(Platform-Specific Incivility) Thinking about the posts and political discussions around the campaign and the Presidential election you witness these days on social media, how likely or unlikely would it be for you to come across the following messages on each of the following social media platforms? Scale – to + : highly unlikely / unlikely / somewhat unlikely / neither likely nor unlikely / somewhat likely / likely / highly likely

(Exclusion) Messages that deny political or social groups the right to participate in politics.

a. Whatsapp
b. YouTube
c. Twitter
d. Facebook
e. Telegram

(Impoliteness) Messages containing name-calling (such as "traitor", "idiot"), offensive and/or pejorative language.

a. Whatsapp
b. YouTube
c. Twitter
d. Facebook
e. Telegram

(Physical Harm/Violence) Messages that threaten others with physical harm or incite others to inflict harm to other individuals.

a. Whatsapp
b. YouTube

c. Twitter
d. Facebook
e. Telegram

(Negativity) Messages that evoke negative emotions such as hatred anger or anxiety.

a. Whatsapp
b. YouTube
c. Twitter
d. Facebook
e. Telegram

(Personal Attack) Verbal attacks to political opponents' and their family members based on their physical characteristics, character or personal beliefs.

a. Whatsapp
b. YouTube
c. Twitter
d. Facebook
e. Telegram

(Stereotype/Hate Speech/Discrimination) Messages negatively associating a person with a specific societal group by using offensive labels related to their sexuality, gender or race.

a. Whatsapp
b. YouTube
c. Twitter
d. Facebook
e. Telegram

(Threat to Democratic Freedoms) Messages threatening democratic freedoms such as proposing to stage a coup to overthrow a democratically-elected government.

a. Whatsapp
b. YouTube
c. Twitter
d. Facebook
e. Telegram

(Online-Offline Incivility) Compared to your political discussions or those discussions you witness outside the Internet, are the political discussions you engage in or witness online:

a. Very negative - Very positive
b. Not respectful at all - Very respectful
c. Not inclusive at all - Very inclusive

d. Very angry - Not angry at all

e. Very intolerant - Very tolerant

f. Very uncivil - Very civil

(Online Political Discussion Experiences) Considering your conversations about politics and the elections on social media and messaging apps, did you experience any of the following situations in the past month? Scale – to +: Never / Less than few times a month / A few times a month / A few times a week / Every day or almost every day

a. Was attacked or insulted by someone else during a political discussion

b. Attacked or insulted someone during a political discussion

c. Felt offended by someone else during a political discussion

d. Witnessed a tense or otherwise uncomfortable political discussion

(Political Self-Censorship) When people have been rude or attacked you online, how often have you responded by? [- Never, Rarely, Sometimes, Often, All of the time +]

a. Telling them to stop

b. Continue the argument and articulate further

c. Leaving the whole conversation

d. Just ignoring the person who attacked you

e. Refrain from participating in other political conversations and/or sharing your political views

f. Making insulting or attacking comments back

g. Unfriended, unfollowed, reported or blocked the person

Section VII. Disinformation Perception

(Perception of Disinformation) How often do you come across political news or information that you suspect is false?

(- Never, Rarely, Occasionally, Frequently, Very often +)

a. On Facebook

b. On Twitter

c. On YouTube

d. On Whatsapp

e. On Telegram

f. On news media (e.g., newspapers, TV, radio, news websites)

Section VIII. Authority Trust

(Trust in Institutions) Please indicate your level of agreement with the following statements:

(1 Fully disagree ... Fully agree 7)

a. I trust the parliament
b. I trust politicians
c. I trust political parties
d. I trust the media
e. I trust the legal system
f. I trust the police
g. I trust the military/Armed Forces
h. I trust the government

Section IX. Populism

(Populism) Please indicate your level of agreement with the following statements.

(1 Fully disagree ... Fully agree 7)

a. Politicians are not really interested in what people like me think.
b. Politicians make decisions that harm the interests of the ordinary people.
c. The people, not the politicians or experts, should make our most important policy decisions.
d. Economic forces and economic interest groups should be brought under greater control.
e. For being truly Brazilian, it is important to have been born here.
f. Achieving compromise among differing viewpoints is important in politics.
g. The will of the majority should be exercised instead of constantly measuring the rights of minorities.

Section X. Attitudes to Democracy

(Support for Democracy) Please indicate how much you agree/disagree with the following statement:

Democracy is the best system for a country like Brazil.

(1 Fully disagree ... Fully agree 7)

(Satisfaction with democracy) On the whole, how satisfied or dissatisfied are you with the way democracy works in Brazil? Are you ...

1. Not at all satisfied
2. Not very satisfied
3. Fairly satisfied
4. Very satisfied

Consent for data linkage

Additionally, we asked the survey respondents to provide their Twitter handles given their consent:

(Twitter handle) Do you have a personal Twitter account?

a. Yes

b. No

(If Yes) We are interested in being able to linking people's answers to this survey to the ways in which they use Twitter. This survey is part of a research project about who and how people use Twitter conducted by a team of researchers at University of Zurich. Are you willing to provide us with your personal Twitter account and for this to be passed to researchers at University of Zurich, along with your answers to this survey? Your Twitter name would not be published. In compliance with Twitter terms of service, we will neither provide any private user's identifying information nor the full text of the tweets used.

a. Yes

b. No

(If Yes) Please enter your Twitter name here: Open Question (Maximum of 100 characters)

The survey participants are sampled by the survey company NetQuest using stratified quota sampling based on a soft quota of national statistics for age, gender, and geographical area (see Table A). The demographic distribution of the final survey participants is reported in Table B. Results from Pearson's chi-squared tests and Mann–Whitney U tests for age, gender, and geographical area indicate that the survey participants and the national electorate are drawn from the same distribution (see Fig B). We reject the null hypothesis when the p-value is smaller than 0.05. Additional demographic variables of the survey participants are presented in Table B.

3. Identification of Political Influencers

We use a heuristic strategy of identifying political influencers from the 57,645 accounts followed by survey respondents. We define political influencers as a composition of both ordinary citizens and celebrities (e.g., politicians, parties, media outlets, journalists, and ordinary opinion leaders) who satisfy two conditions: 1) have a comparatively large followers base and 2) are likely to produce political content [1, 2]. According to this definition, we select political influencers in three steps. Firstly, we identify influential accounts that have a number of followers exceeding a certain threshold. We experiment with various thresholds and find that more generous thresholds can reveal significant

multi-level clusters, but we also avoid using a threshold that is too low. We choose 1,000 as the ideal threshold for achieving this goal. Fig D (A) displays the Complementary Cumulative Distribution Function (CCDF) plots for the number of followers of accounts followed by survey respondents. We establish a threshold of 1,000 followers, and only accounts exceeding this threshold are retained, accounting for 63% of the total accounts who are followed by survey respondents.
Second, from the accounts with more than 1,000 followers, we further select Brazilian accounts based on the location information displayed in their profile (Brazil or Brazilian cities).
Third, we filter the accounts that might produce political information from the Brazilian influencers, covering categories of politicians, parties, media outlets, journalists, and ordinary opinion leaders. They are also popular categories used in other studies [3–5]. We manually examine approximately 2,000 random profile examples and create a politically relevant keyword list based on these samples. Accounts potentially generating political content are identified by matching politics-related keywords in their profiles and supplemented by additional lists of politicians (based on 2022 presidential election candidates), parties, and media outlets (based on Digital News Report 2022 produced by Reuters Institute Oxford). See Table C for more details.
The three steps result in identifying 2,307 Brazilian political influencers from the 57,645 followed accounts. Moreover, out of 271 individuals, 204 survey participants are found to follow political influencers. We examine the distribution of demographic variables among these 204 respondents and compare it to the demographic variables of 1,018 survey respondents (see Fig C). The Pearson's chi-squared test and Mann–Whitney U test results across all variables show that the null hypothesis of the same distribution between our sample and survey respondents cannot be rejected. Therefore, based on our evidence, this 204-person subsample does not introduce significant bias into either the overall group of survey respondents or the national electorate.

4. Multi-dimension Annotation of Identities of Political Influencers

The annotation results on Political Ideology, Campaign Support, Social Identity, and Account Type for influencers in communities of all scale levels are displayed in Fig E, Fig F, Fig G, and Fig H.

5. Multi-scale Community Detection

At the heart of the multi-scale community detection method proposed by [6] lies the generalized Markov Stability function, which quantifies the quality of a partition H of a graph G at a specific time scale t. The goal is to find partitions that maximize this stability, indicating strong community structures.
The optimization problem is formulated as:

$$H^*(t) = \arg\max_H Q_{\text{gen}}(t, H) = \arg\max_H \operatorname{Tr}\left[H^\top \left(F(t) - \sum_{k=1}^{m} v_{2k-1} v_{2k}^\top \right) H \right]$$

Where:

- $H \in \mathbb{R}^{N \times c}$ is the indicator matrix for the partitioning of N nodes into c communities.
- $F(t) \in \mathbb{R}^{N \times N}$ is the node similarity matrix at time t, capturing the probability of a random walker transitioning between nodes over time.
- $\{v_k\}_{k=1}^{2m}$ are vectors defining the null model, representing expected connections in a randomized version of the graph.

This formulation ensures that the detected communities are not only cohesive but also statistically significant when compared to a null model.
The parameter t serves as a resolution factor:

- Small t: The random walker has limited time to move, leading to finer community structures.
- Large t: The random walker explores more of the graph, revealing coarser community structures.

By analyzing the stability of partitions across different t values, it uncovers the multiscale community structure inherent in complex networks.
This method finds communities as groups of nodes that best retain the flow of a random walk process. Longer times used by the random walker correspond to larger scales. Optimal scales are found as minima of the variation of information of an ensemble partitions computed at each scale [7]. The process results in seven optimal scales in the Brazilian network. We discard the two first scales with 1767 and 1170 communities, respectively, as they have very low granularity, and more than 90 percent of the communities only include one node. Finally, we have five levels for analysis, with 46, 14, 8, 3, and 2 communities respectively.
For each scale s, the community detection yields a partition of the influencer network $\mathcal{G}^{\mathcal{S}}$ in K_s non-overlapping communities $\{C_i^s\}_{i=1}^{K_s}$. By projecting the partitions on the consumer network $\mathcal{G}^{\mathcal{C}}$, we obtain, possibly overlapping, communities of consumers where two nodes are in the same community if they follow influencers belonging to the same community. This overlapping partitioning of the consumer network can also be seen as a hypergraph, where for each community C_i^s at scale s in the influencer network, corresponds a hyperedge, E_i^s in the consumer network defined as the set of users following at least one influencer in C_i^s. At scale s, $\{E_i^s\}_{i=1}^{K_s}$ is therefore the set of K_s hyperedges of the consumer network mapping the community partition $\{C_i^s\}_{i=1}^{K_s}$ of the influencer network.
The community detection in the influencer network is implemented with the Python package *PyGenStability*[1], which is designed for multi-scale community detection with Markov Stability [8] and includes an automatic detection of significant scales.
We run the code with the following parameters *PyGenStability*:

```
method = "leiden",
min_scale=-3,
max_scale=3,
n_scale=1000,
n_tries=100,
constructor= "linearized",
n_workers=4,
```

For more information about the parameters, please refer to the documentation.
The result of *PyGenStability* multi-scale community detection is shown in Fig D (B). The Markov time t (resolution parameter) is varied from 10^{-3} to 10^{3}, with 1000 steps, and robust partitions are detected as minima of the Normalized Variation of Information (NVI) of an ensemble of 100 partitions computed at each step. We obtain seven robust scales, and five are manually evaluated as effective partitions.

6. Evaluation of Measurements for Selective Exposure Indices

Identity Diversity

The index of Identity Diversity is calculated for each community and is based on the labels assigned to political influencers. As introduced in section 3, we annotate political influencers on four dimensions:

[1] `https://barahona-research-group.github.io/PyGenStability/`

Political Ideology, Campaign Support, Social Identity, and Account Type. We use Political Ideology labels as the main indicator of the political identity of political influencers since they are the most commonly used labels in politics. This approach also simplifies inferring the political identity of accounts with missing labels, as they can be assigned probabilities from the three options: {Left, Right, Center}.

As we only label accounts that explicitly reveal their ideology on their profile, we have several accounts in each community that are unlabeled. We put forward two approaches to deal with the unlabeled values when calculating the diversity of Political Ideology of political influencers with the Gini-Simpson Index: First, we do not consider the unlabeled accounts and, second, we compute the Gini-Simpson Index, which is the probability of drawing two labels from a community that are different from one another, by assigning probabilities of political ideology labels to the unlabeled accounts equal to the proportions of political ideology labels in the community. Using both strategies in the regression analysis did not significantly change the results.

Information Diversity

The index of Information Diversity is calculated on each community and based on the website domain links posted by the political influencers. We unshorten the website links and extract the domain from the URLs with the Python package *unshortenit*[2], and calculate the diversity of domains using the Gini-Simpson Diversity Index. Communities with fewer than 100 shared domain links or where less than 50% of the influencers share domain links are excluded due to reliability concerns (see Fig I).

7. Statistical Regression Modeling

The regression modeling between the individuals' attributes (independent variables) and selective exposure indices (dependent variables) consists of three steps:

First, we reduce the dimensions of the 189 independent variables obtained from ten thematic groups of survey questionnaires: Demographics, News Consumption, Political Communication, Political Identification, Political Engagement, Perceptions of Incivility, Perceptions of Disinformation, Authority Trust, Populism, and Attitudes toward Democracy (see section 2). PCA projection is implemented to observe the clusters or sub-dimensions formed by these variables. We define the sub-dimensions heuristically by combining the results from both the Biplot (Fig J) and Screeplot (Fig K). All the sub-dimensions have been qualitatively interpreted (see Table D). The variables included in each sub-dimension are shown in Table E. We choose one representative variable from each sub-dimension for the following regression analysis. Finally, 25 variables are selected after the dimension reduction.

Second, as the values of Community Overlap are positive integers, we choose a zero-truncated negative binomial regression model, implemented with the *vglm*[3] package in R and the family is set with *posnegbinomial.* The other indexes take values in the real numbers between 0 and 1. We use a beta regression model implemented with the *betareg* in R due to the flexibility of modeling diversity and concentration indices in the unit interval [9, 10]. To deal with zeros and ones values in the indices, we utilize the following transformation approach from Ref. [9], which transforms the variable $y \in [0, 1]$ to $y' \in (0, 1)$

$$y' = \frac{y(N-1) + \frac{1}{2}}{N}, \tag{1}$$

where N is the sample size. We discard Level 5 because we only have values for 2 communities, and the communities detected at Level 5 have a similar pattern to Level 4.

[2] `https://pypi.org/project/unshortenit/`

[3] `https://www.rdocumentation.org/packages/VGAM/versions/1.1-11/topics/vglm`

Third, we select the optimal regression models using Bayesian Information Criterion (BIC) forward selection with the reduced 25 variables. BIC forward selection is a statistical method used for model selection in regression analysis. Forward selection is a greedy optimization approach to find the best subset of predictors that results in the most parsimonious model with a good balance between fit and complexity. The lower the BIC values, the better the model fits the data. The rationale of forward selection is that we start with no predictors in the null model and add variables individually based on their contribution to reducing the BIC. The procedure stops when adding any remaining predictor does not lower the BIC value. The BIC evaluation for regression models of the five selective exposure indices (including six measurements because we use two approaches for the measurement of Identity Diversity) from Level 1 to Level 4 can be found in Figs. L, M, N & O. We only keep the variables that decrease BIC values in the final regression models.
To validate the model fit, the distribution of predicted values obtained from the regression models are compared with the actual distribution of values of the six measurements (see Fig P, Fig Q, Fig R, Fig W). The alignment of fitted distributions and the real value distributions demonstrate a good model fit.
The residual distributions for the results of Beta Regression are examined at the four scale levels (see Fig T, Fig U, Fig V, Fig W). Additionally, we conduct a sensitivity check by manually removing outliers that cause the simulated line to deviate from 0 and then rerun the regression. The residual distributions after removing outliers are shown in Fig X, Fig Y, Fig Z, Fig AA. The regression plot after removing outliers is displayed in Fig AB. Even though there are small changes in the coefficients, the signs of all the coefficients remain unchanged (except for the regression model of Structural Integration at scale level 4, which does not converge when outliers are removed). This indicates that the results are not biased by outliers.

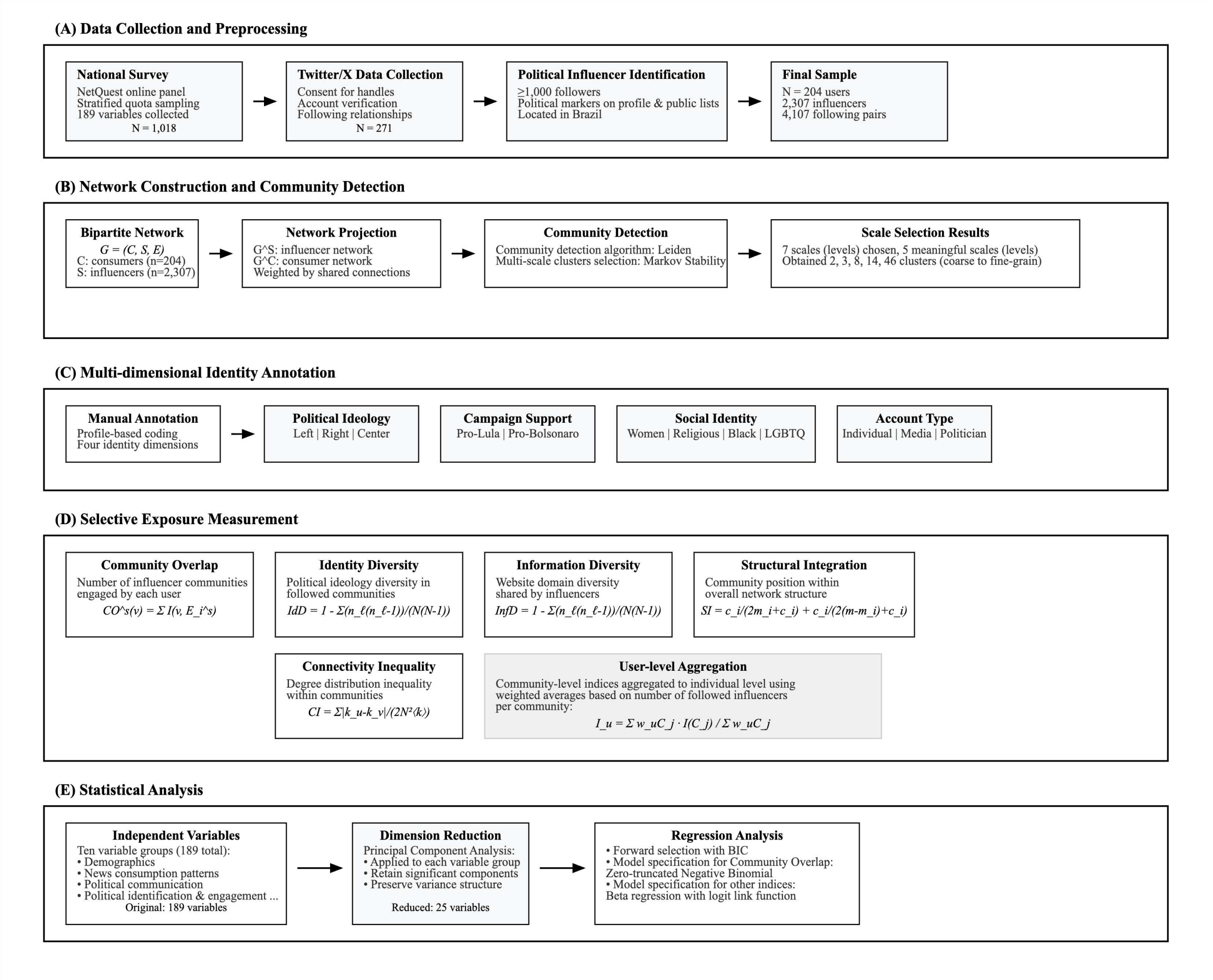

Fig A: Flowchart of the Methodological Design Overview. The analytical framework comprises five stages: (A) Data Collection and Preprocessing. (B) Network Construction and Community Detection. (C) Multi-dimensional Identity Annotation. (D) Selective Exposure Measurement. (E) Statistical Analysis.

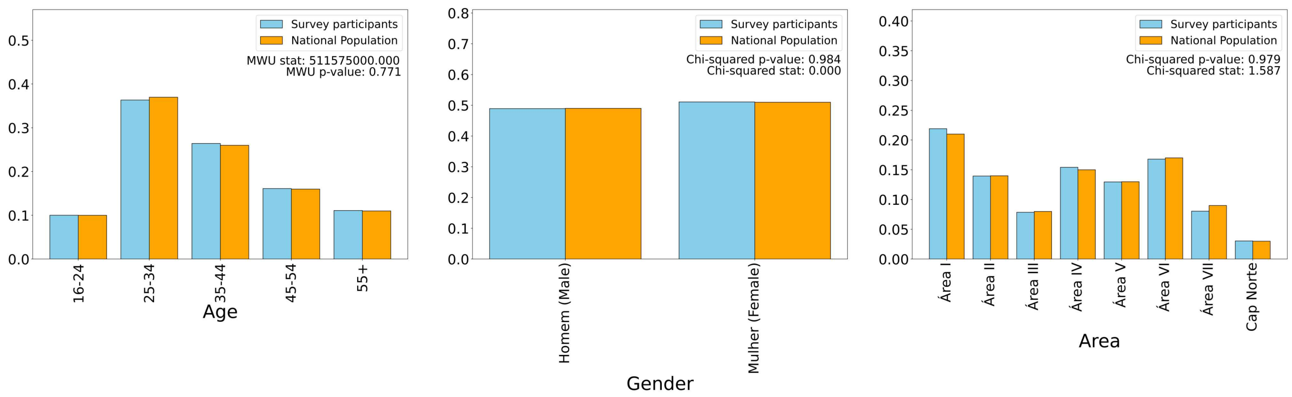

Fig B: Comparison of distributions on Gender, Age, and Area between survey participants and national population. Pearson's chi-squared test is applied for categorical variables, including Gender and Area. Mann–Whitney U test is applied for discrete variables, including Age. The national population statistics are provided by the survey company NetQuest as soft quotas.

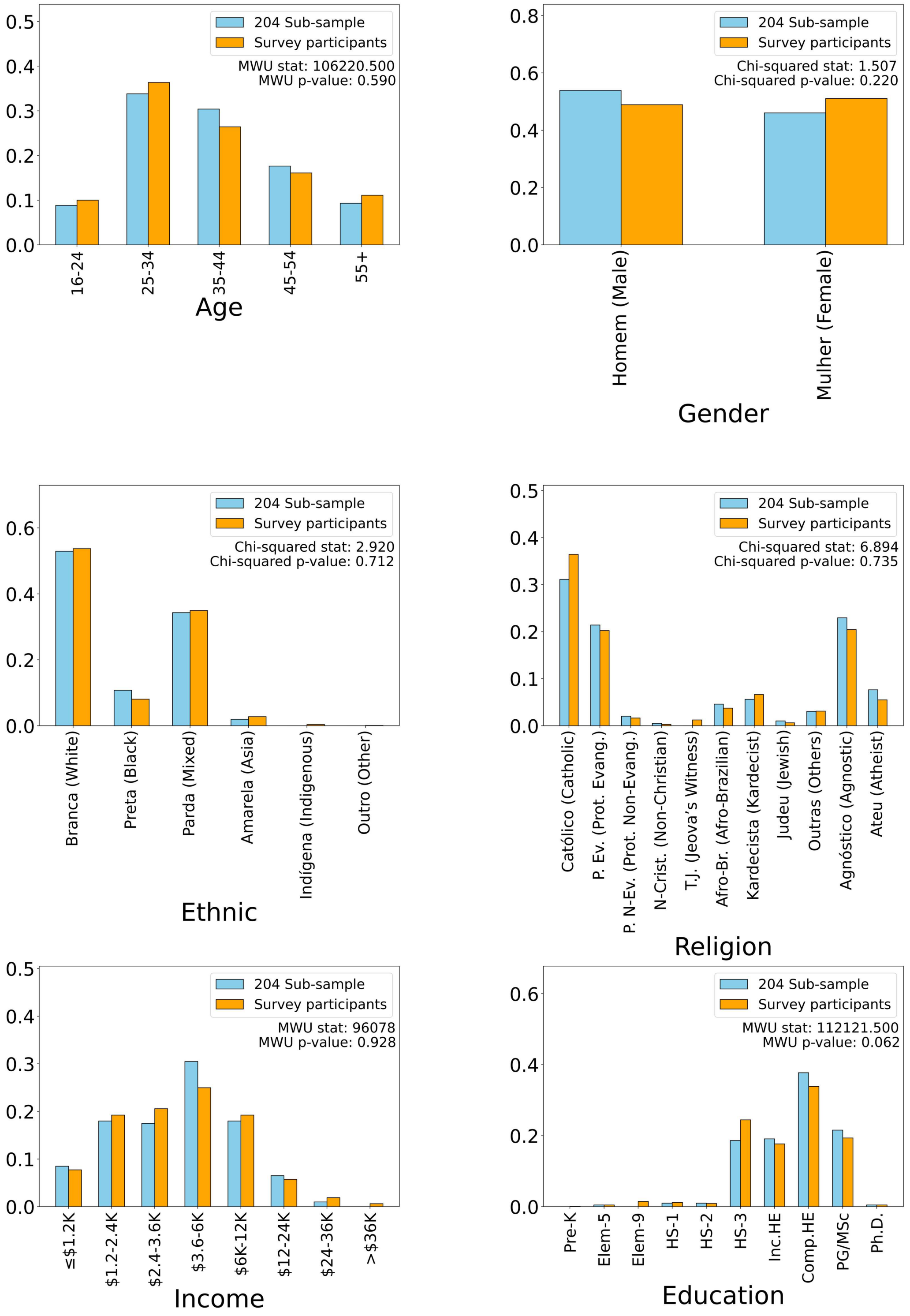

Fig C: Comparison of distributions on Age, Gender, Ethnic, Religion, Income, and Education between survey respondents (N = 1,018) and its sub-samples who follow political influencers (N = 204). Pearson's chi-squared test is applied for categorical variables, including Gender, Ethnic, and Religion. Mann–Whitney U test is applied for discrete variables, including Age, Income, and Education.

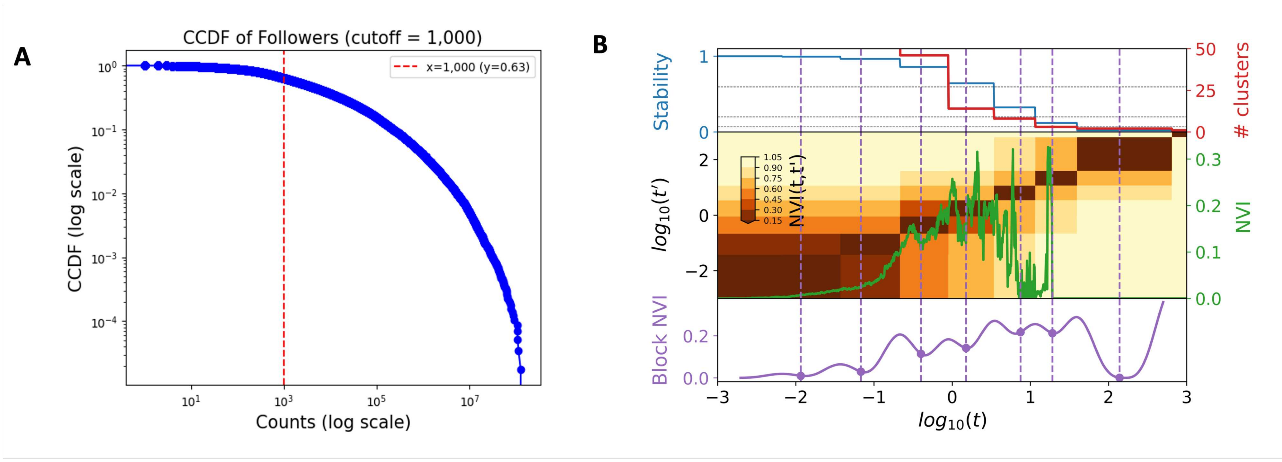

Fig D: (A) displays the complementary cumulative distribution function (CCDF) plots of the number of followers of Twitter accounts followed by survey respondents, with cutoffs at 1,000. (B) shows the corresponding multi-scale community detection outcomes at different scales.

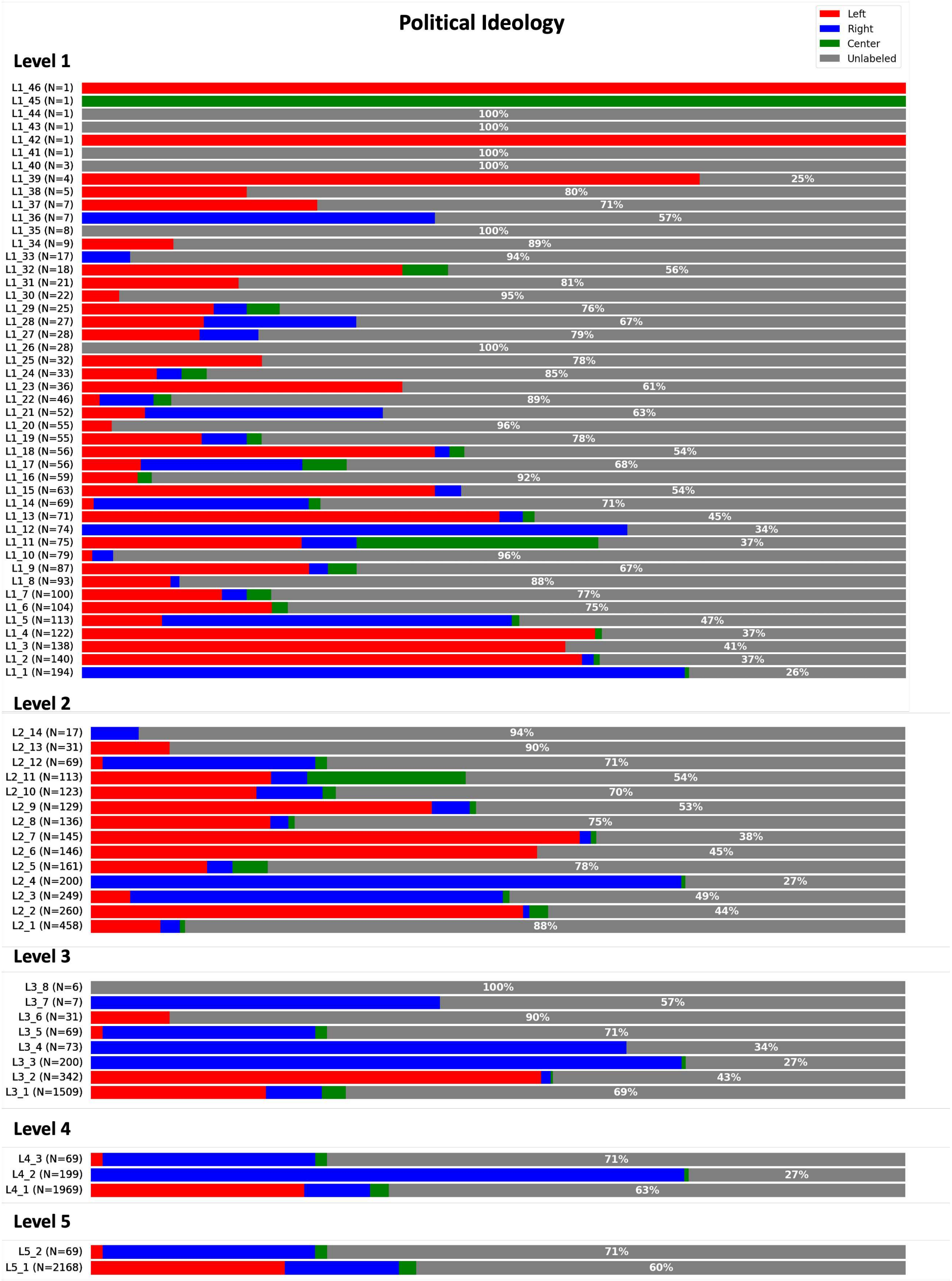

Fig E: Annotation of political ideology among political influencers in communities across five scale levels.

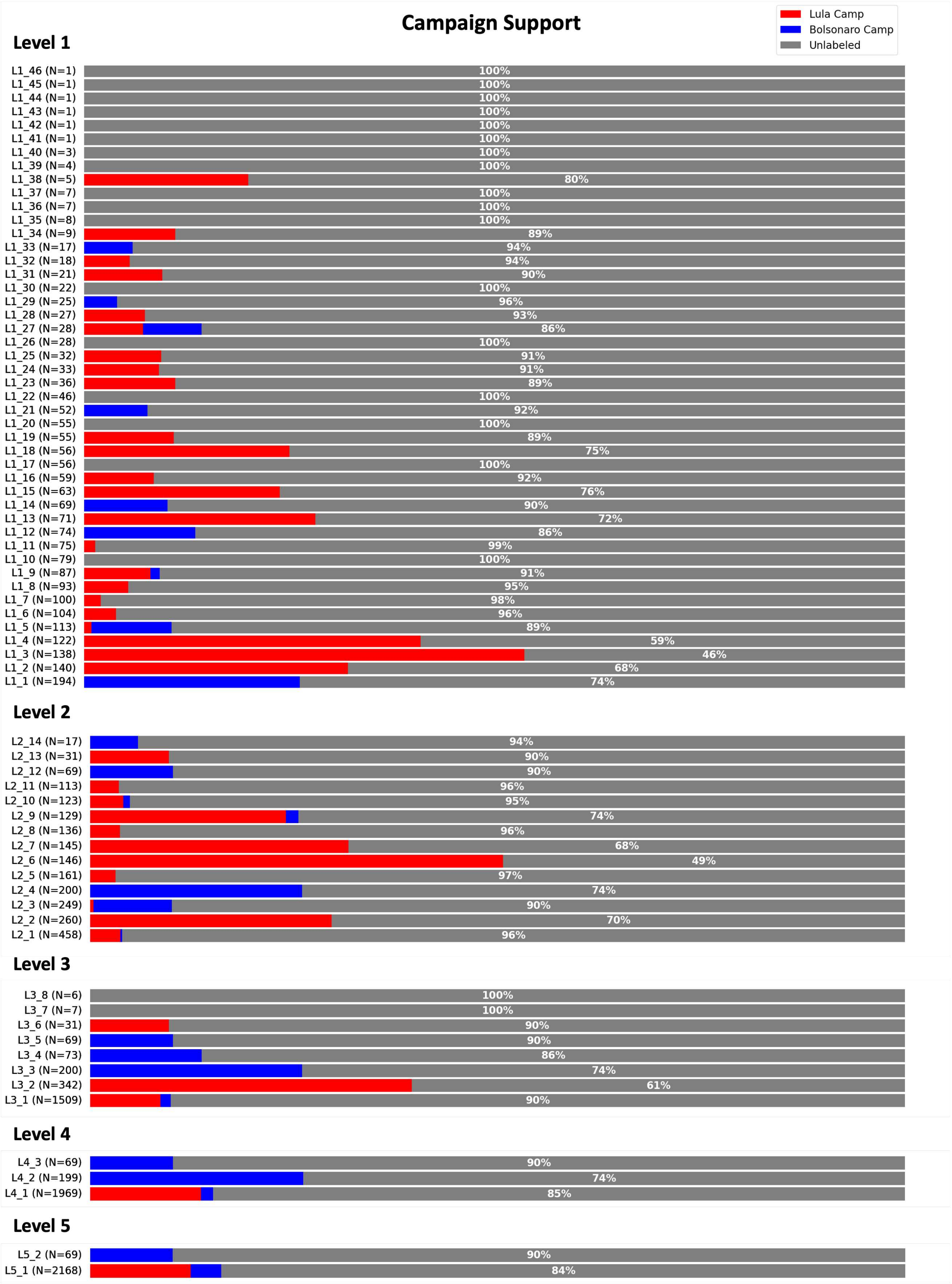

Fig F: Annotation of campaign support among political influencers in communities across five scale levels.

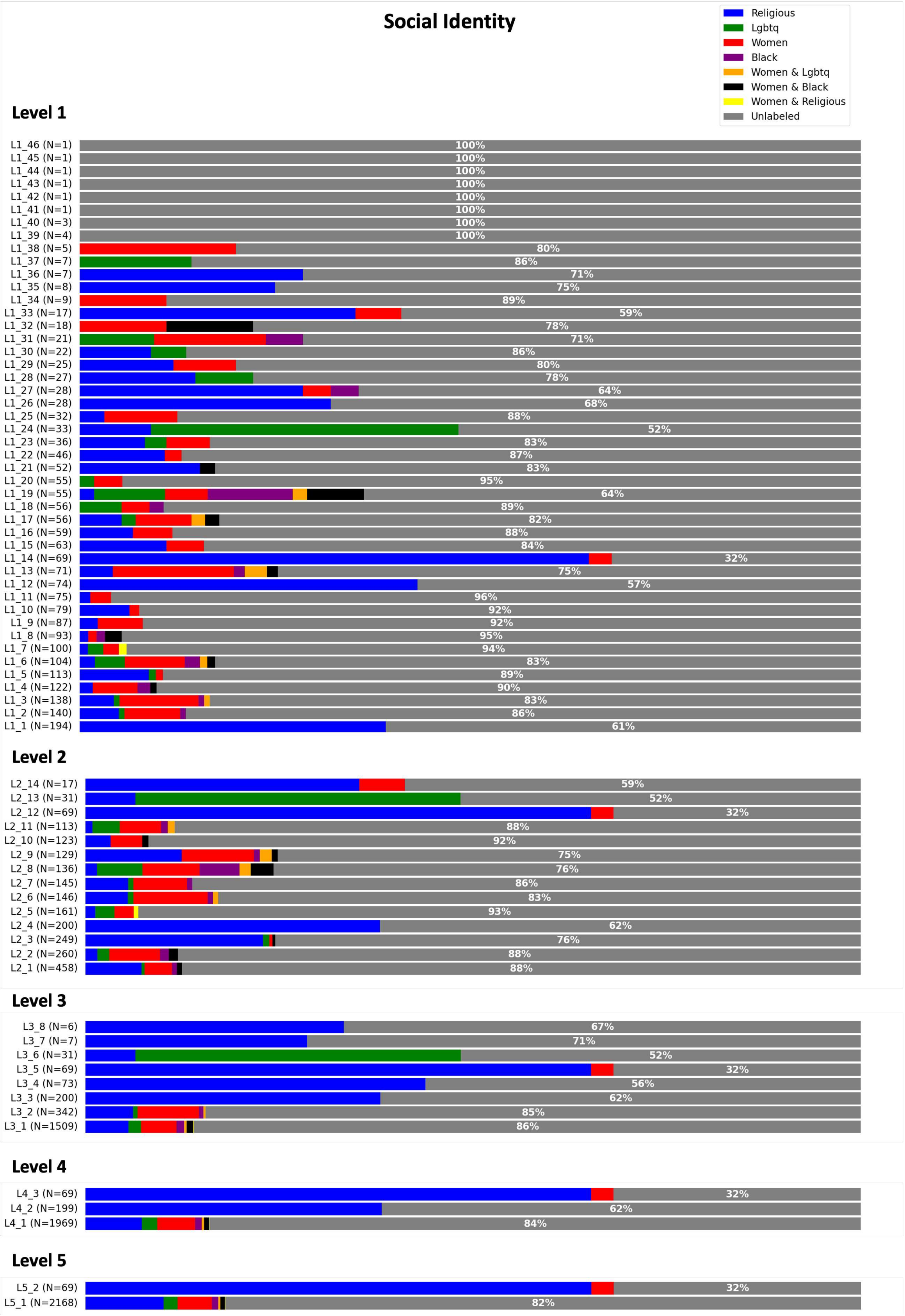

Fig G: Annotation of social identity among political influencers in communities across five scale levels.

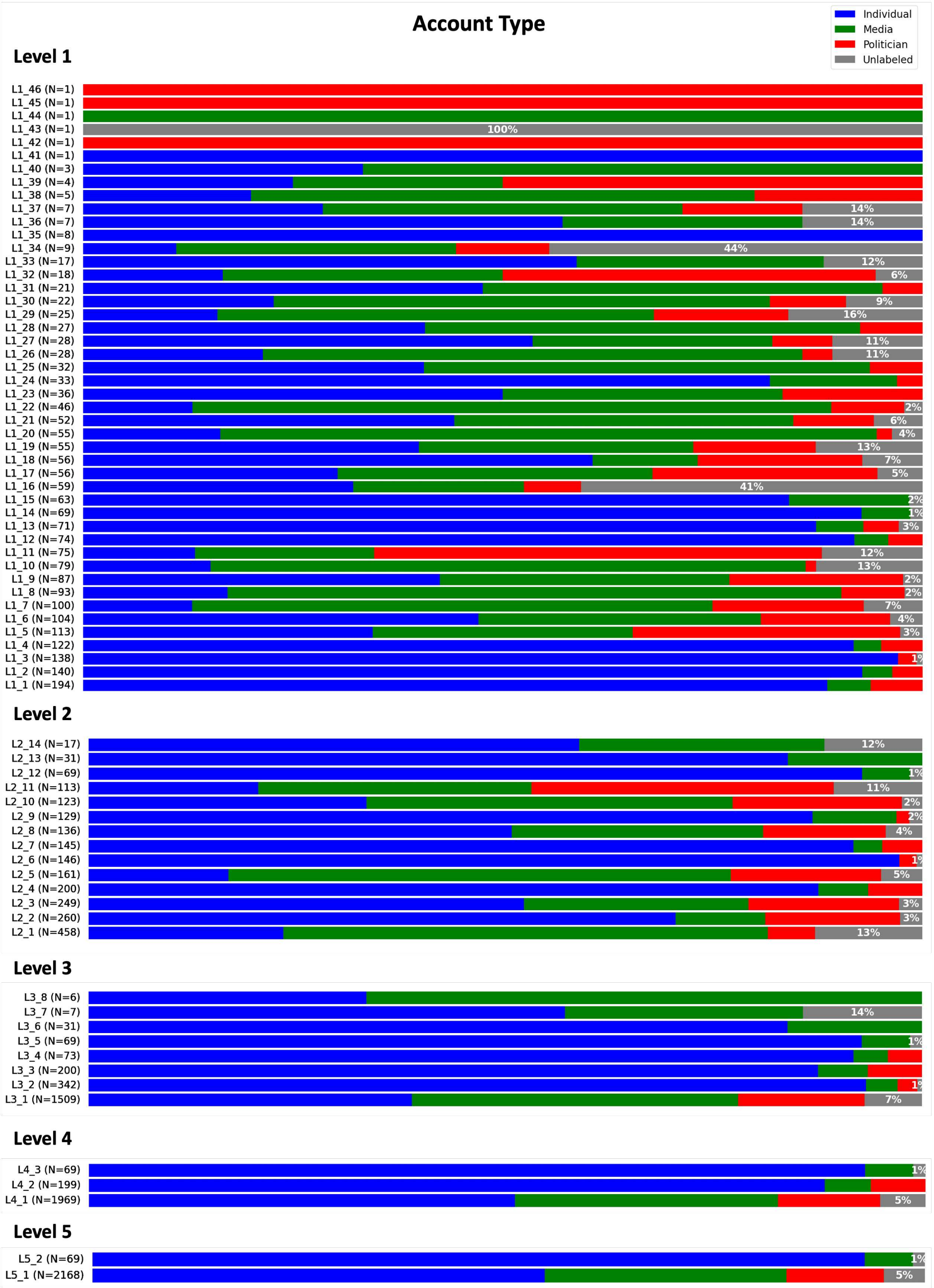

Fig H: Annotation of account type among political influencers in communities across five scale levels.

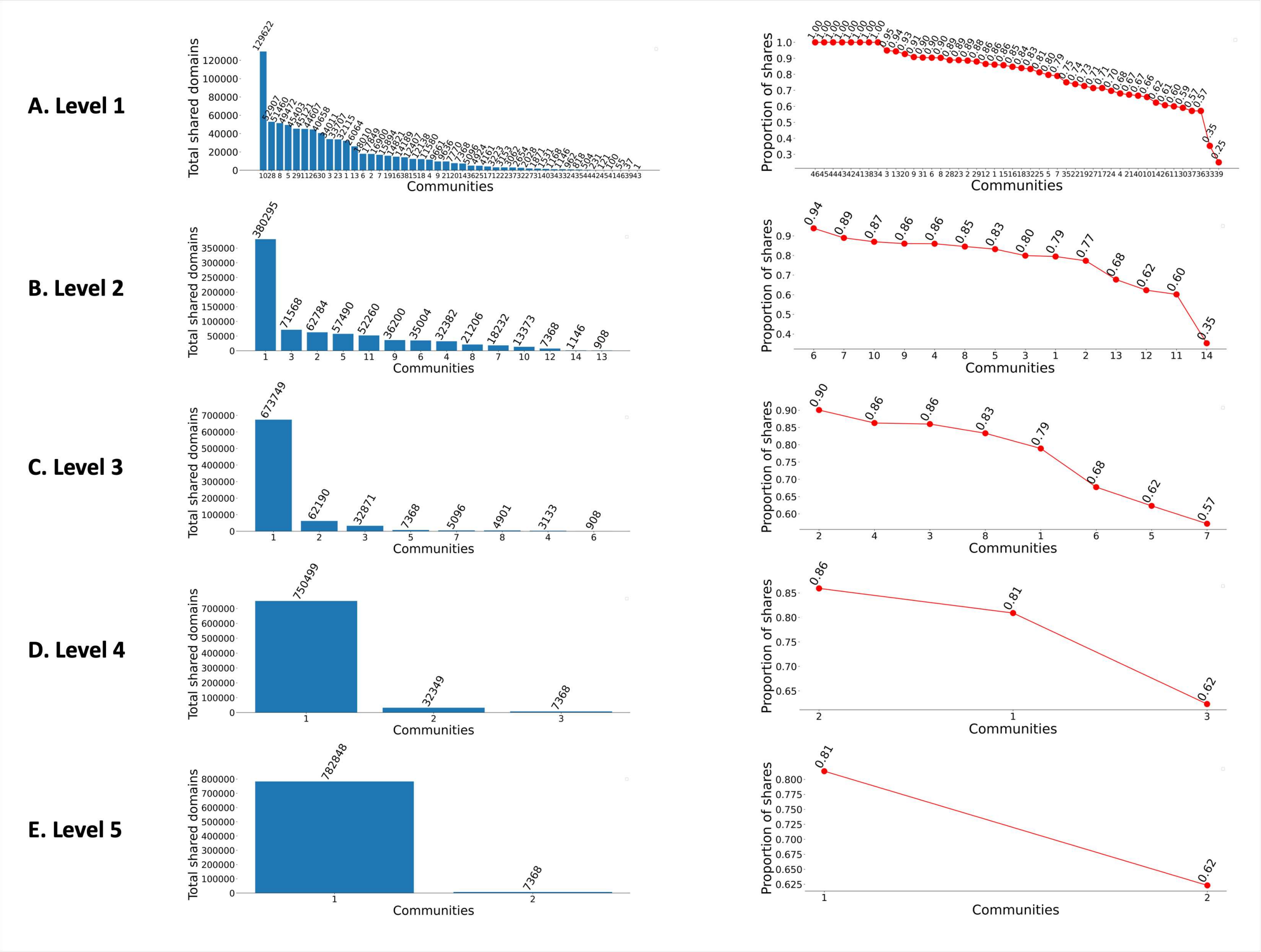

Fig I: The left column shows the total number of website domain links shared by political influencers in communities at (A) Level 1, (B) Level 2, (C) Level 3, (D) Level 4, (E) Level 5. The right column shows the proportion of website domain links shared by political influencers in communities at (A) Level 1, (B) Level 2, (C) Level 3, (D) Level 4, (E) Level 5.

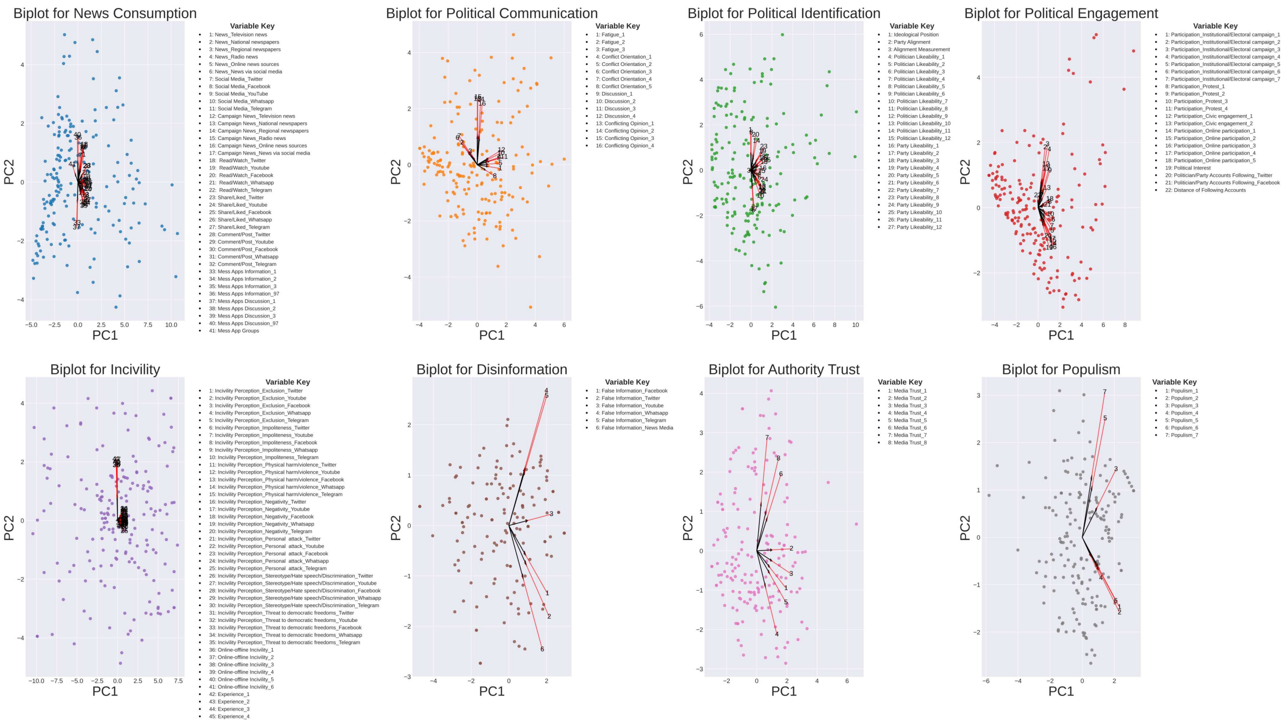

Fig J: Biplot of PCA projection of survey individuals' attribute variables. Clusters are shown within each pre-defined group: News Consumption, Political Communication, Political Identification, Political Engagement, Incivility, Disinformation, Authority Trust, and Populism.

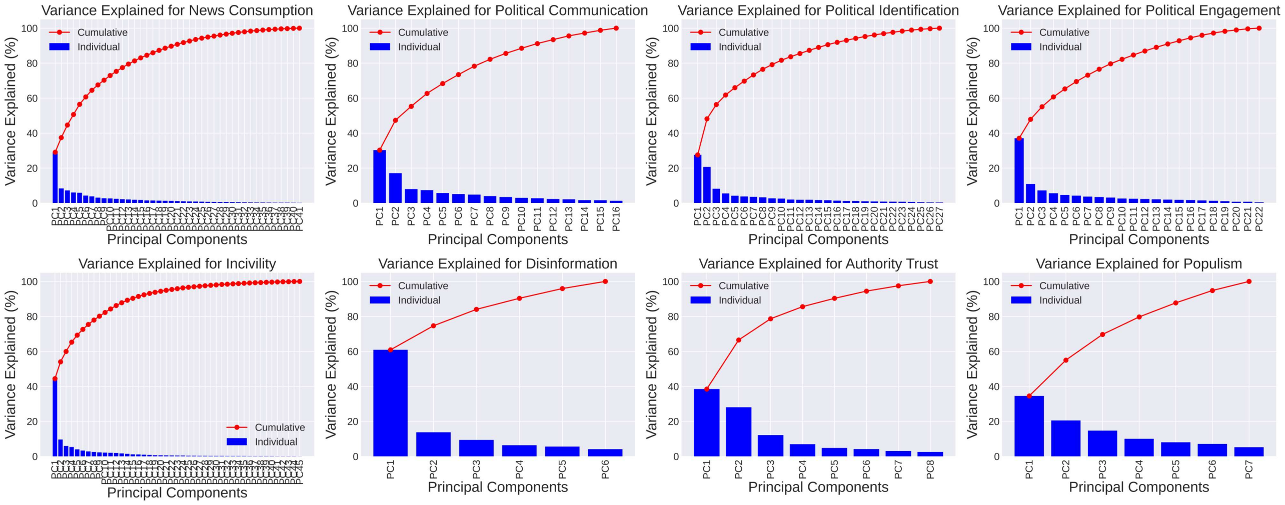

Fig K: Scree plot of PCA projection for survey individuals' attribute variables. The percentages of explained variance are displayed at different numbers of components.

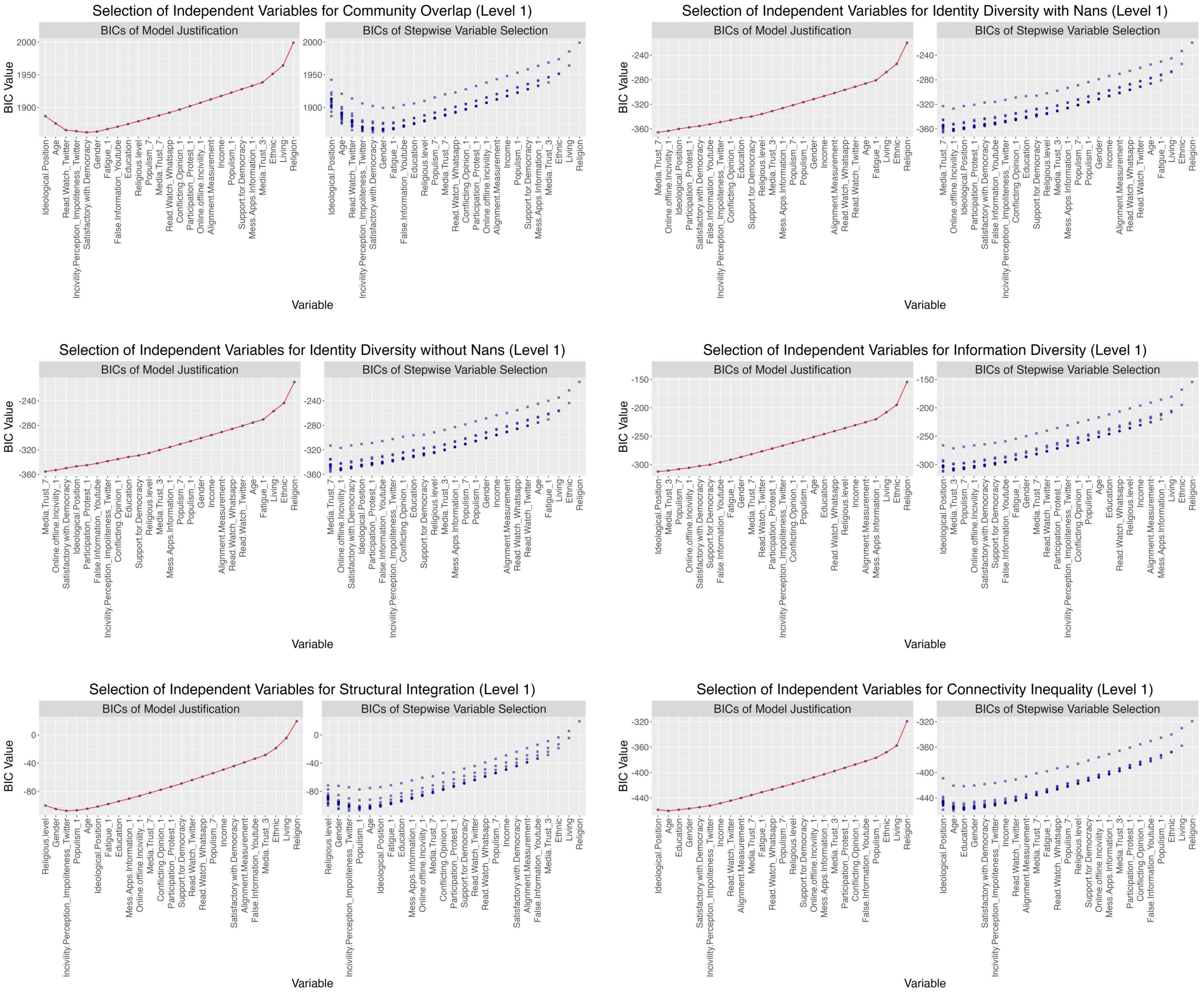

Fig L: BIC forward selection for regression models of six measurements: Community Overlap, Identity Diversity with unlabeled accounts, Identity Diversity without unlabeled accounts, Information Diversity, Structural Integration, and Connectivity Inequality, at scale level 1.

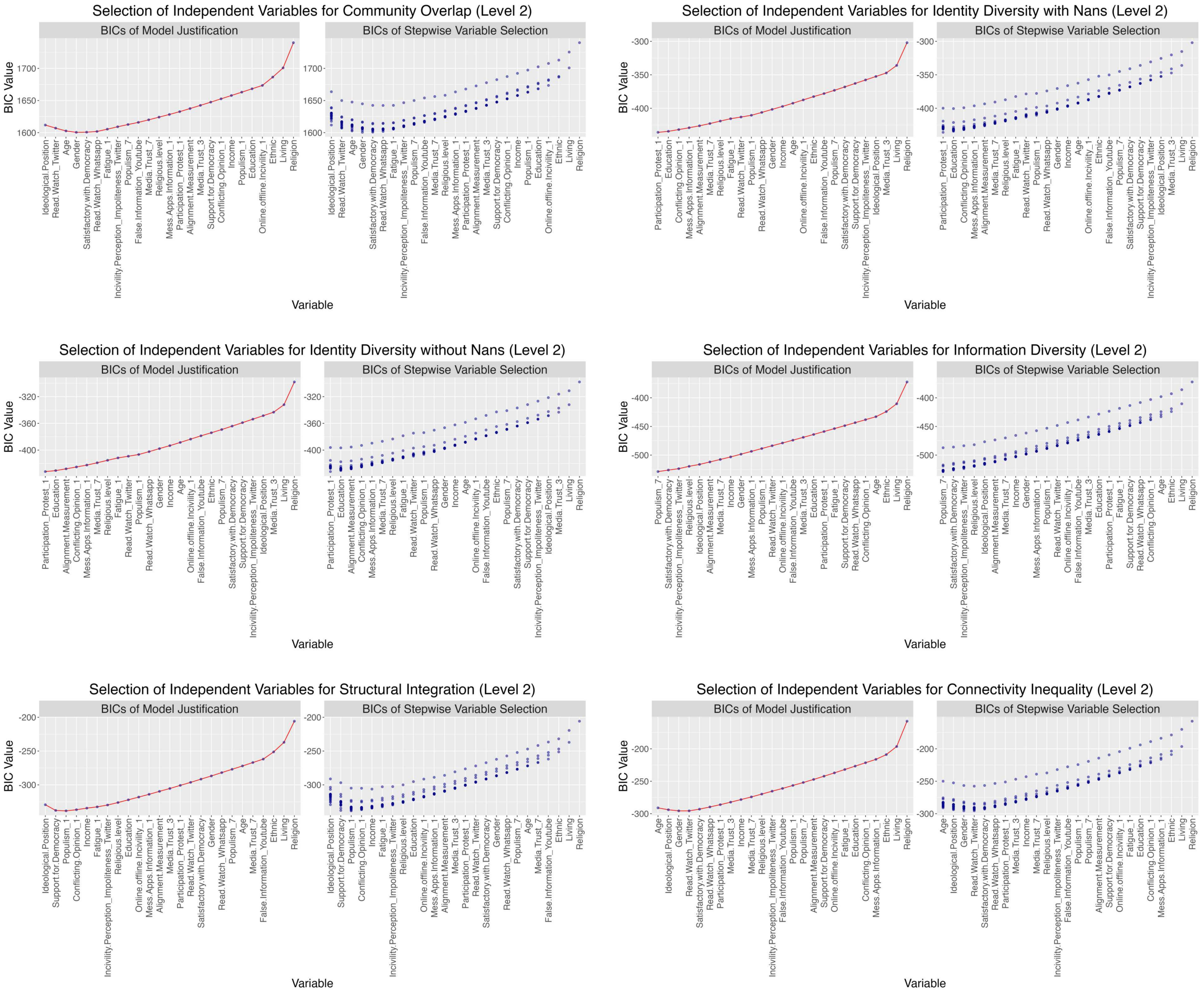

Fig M: BIC forward selection for regression models of six measurements: Community Overlap, Identity Diversity with unlabeled accounts, Identity Diversity without unlabeled accounts, Information Diversity, Structural Integration, and Connectivity Inequality, at scale level 2.

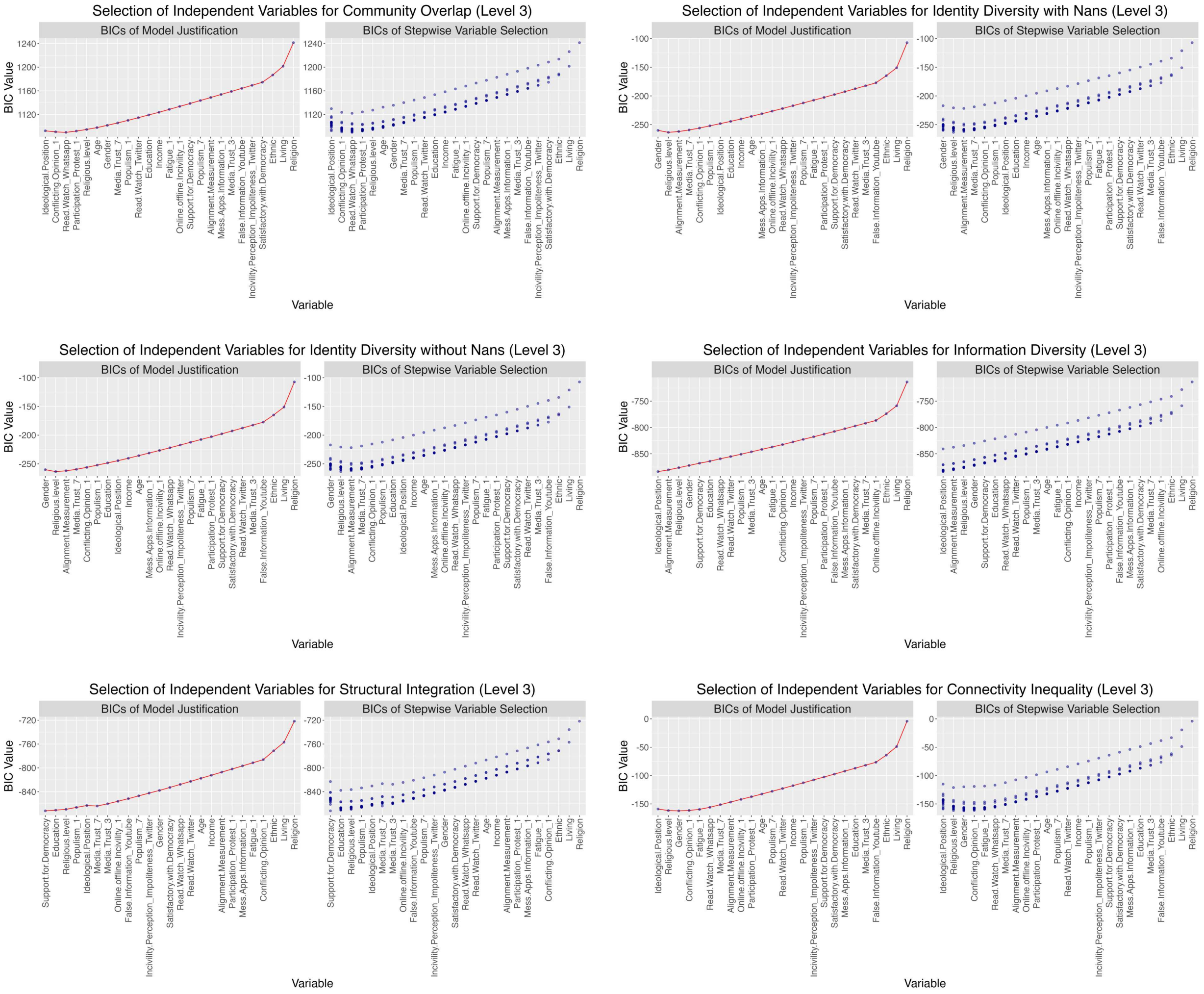

Fig N: BIC forward selection for regression models of six measurements: Community Overlap, Identity Diversity with unlabeled accounts, Identity Diversity without unlabeled accounts, Information Diversity, Structural Integration, and Connectivity Inequality, at scale level 3.

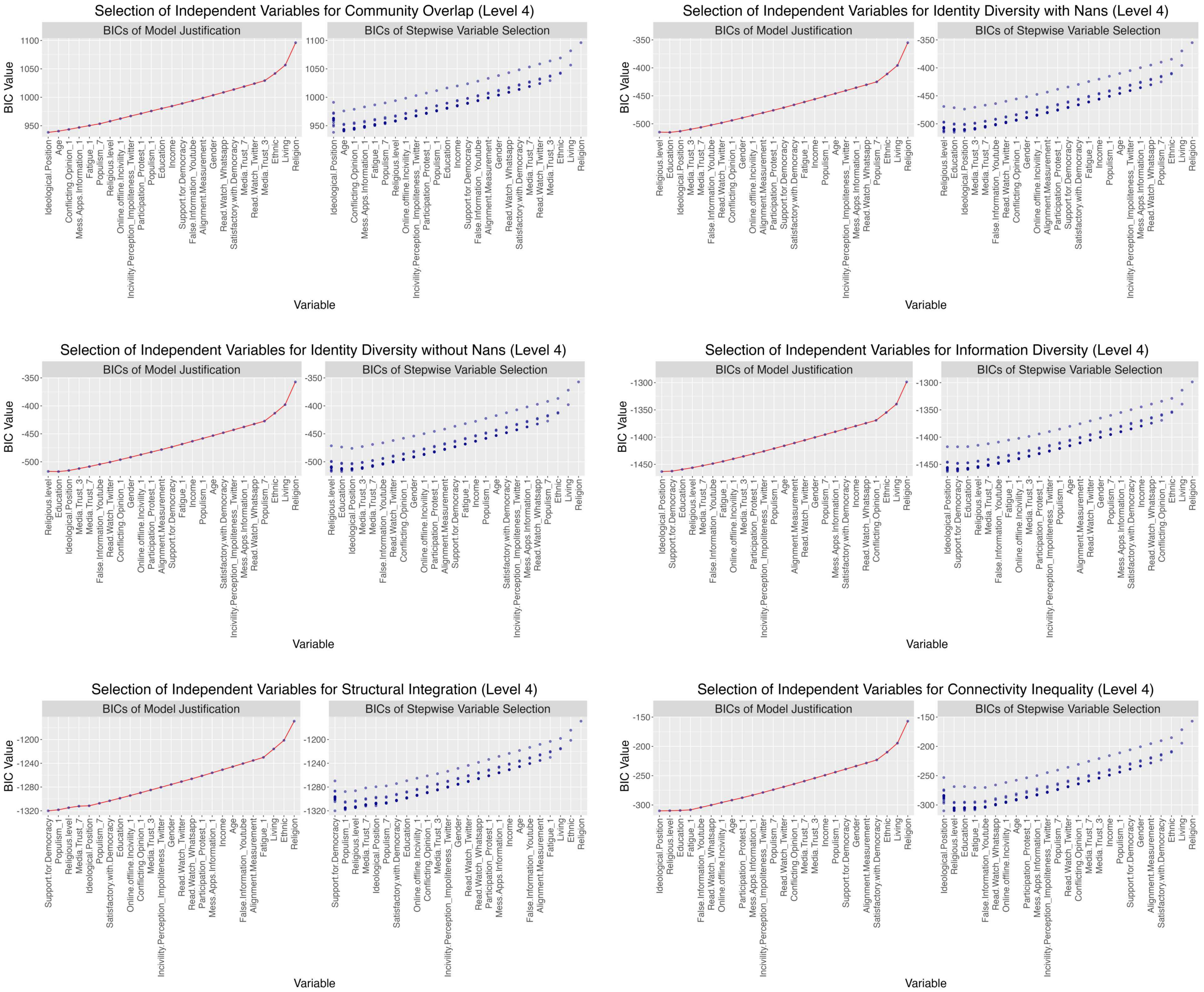

Fig O: BIC forward selection for regression models of six measurements: Community Overlap, Identity Diversity with unlabeled accounts, Identity Diversity without unlabeled accounts, Information Diversity, Structural Integration, and Connectivity Inequality, at scale level 4.

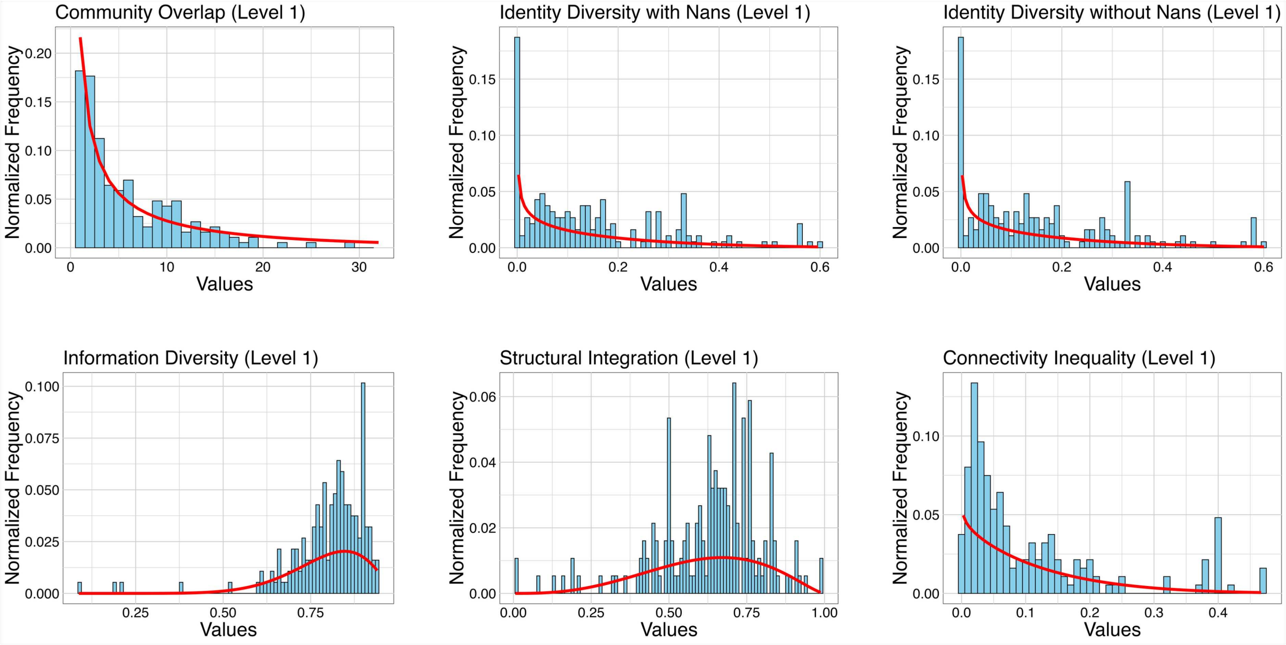

Fig P: Comparison of data distributions between original values and estimated values of the six measurements: Community Overlap, Identity Diversity with unlabeled accounts, Identity Diversity without unlabeled accounts, Information Diversity, Structural Integration, and Connectivity Inequality, at scale level 1.

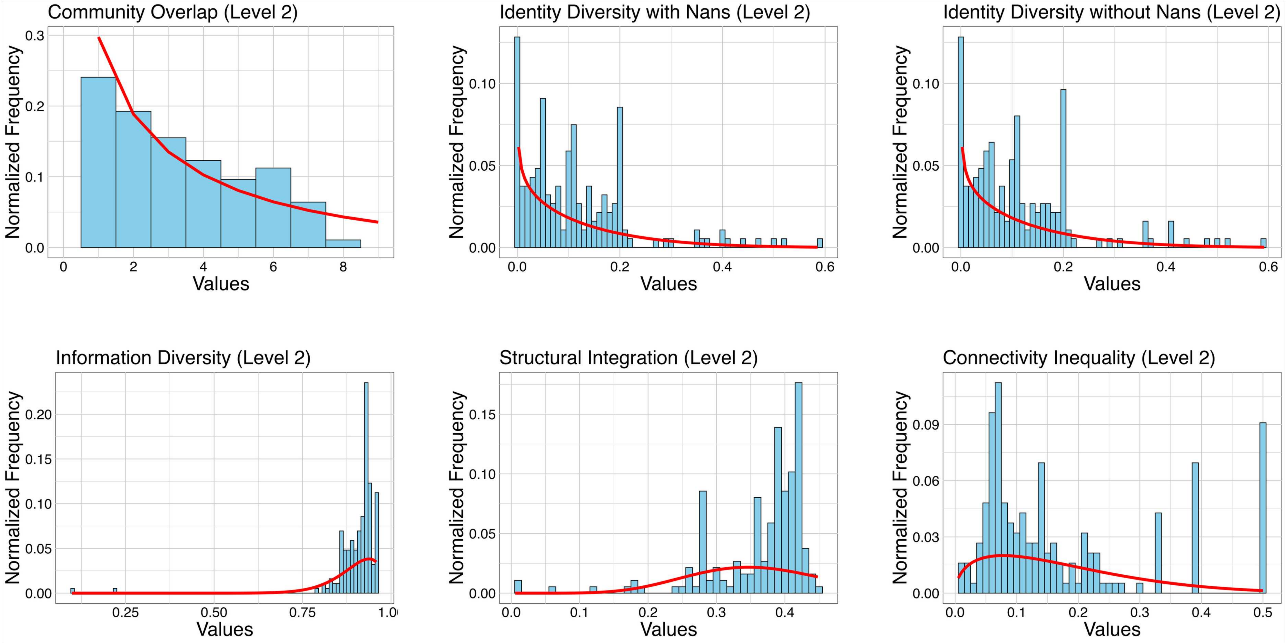

Fig Q: Comparison of data distributions between original values and estimated values of the six measurements: Community Overlap, Identity Diversity with unlabeled accounts, Identity Diversity without unlabeled accounts, Information Diversity, Structural Integration, and Connectivity Inequality, at scale level 2.

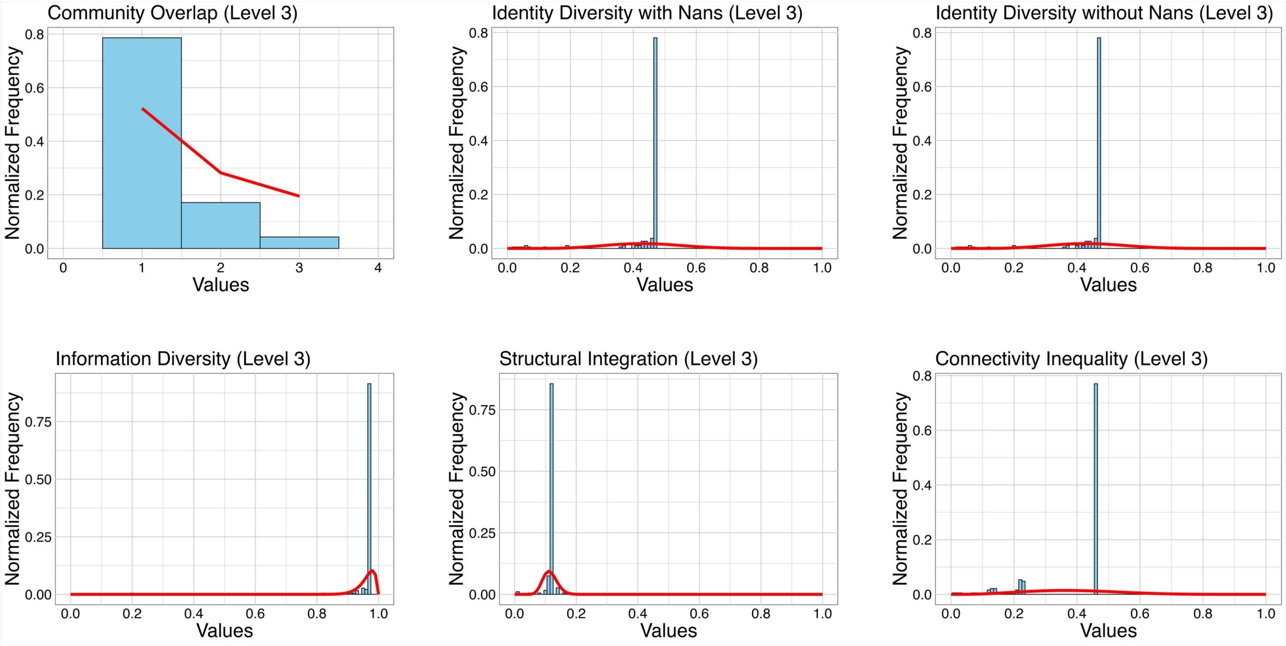

Fig R: Comparison of data distributions between original values and estimated values of the six measurements: Community Overlap, Identity Diversity with unlabeled accounts, Identity Diversity without unlabeled accounts, Information Diversity, Structural Integration, and Connectivity Inequality, at scale level 3.

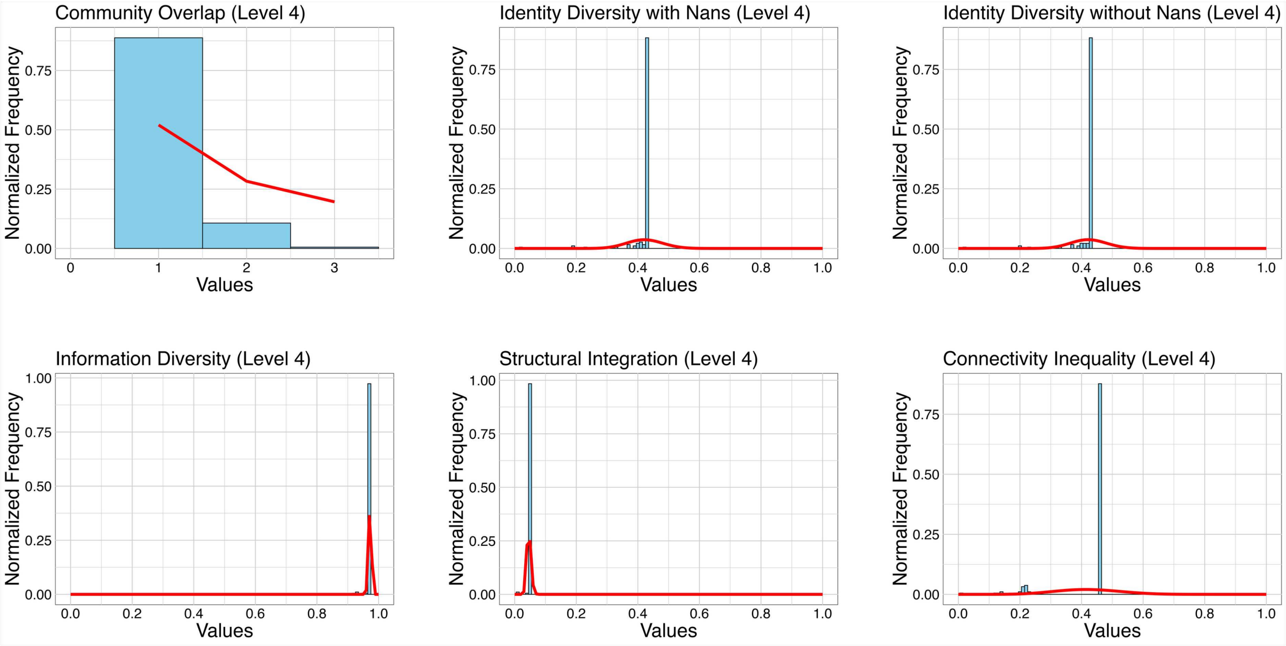

Fig S: Comparison of data distributions between original values and estimated values of the six measurements: Community Overlap, Identity Diversity with unlabeled accounts, Identity Diversity without unlabeled accounts, Information Diversity, Structural Integration, and Connectivity Inequality, at scale level 4.

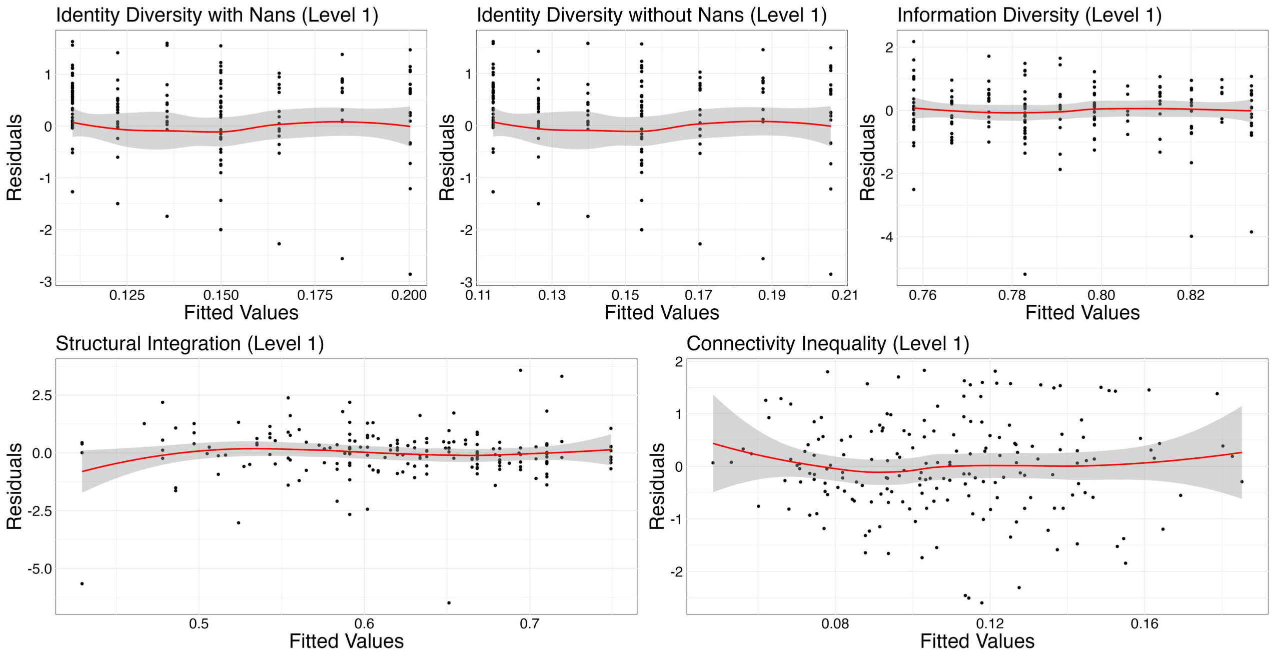

Fig T: Residual check of Beta regression models of five measurements: Identity Diversity with unlabeled accounts, Identity Diversity without unlabeled accounts, Information Diversity, Structural Integration, and Connectivity Inequality, at scale level 1.

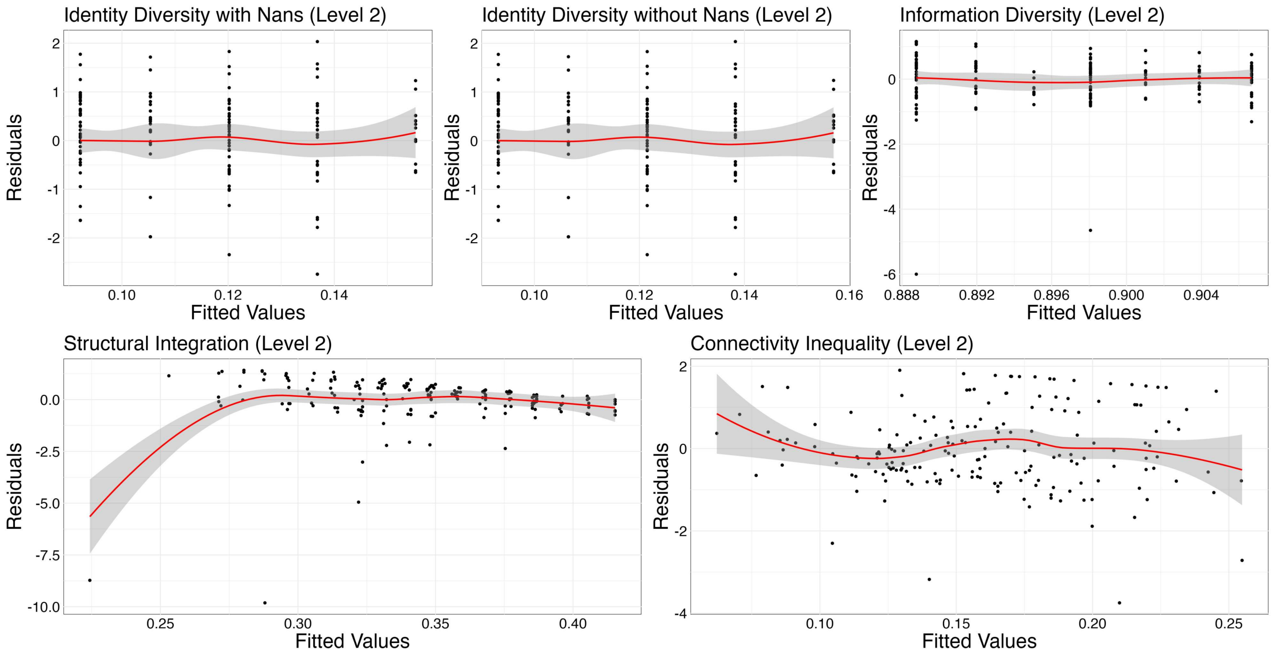

Fig U: Residual check of Beta regression models of five measurements: Identity Diversity with unlabeled accounts, Identity Diversity without unlabeled accounts, Information Diversity, Structural Integration, and Connectivity Inequality, at scale level 2.

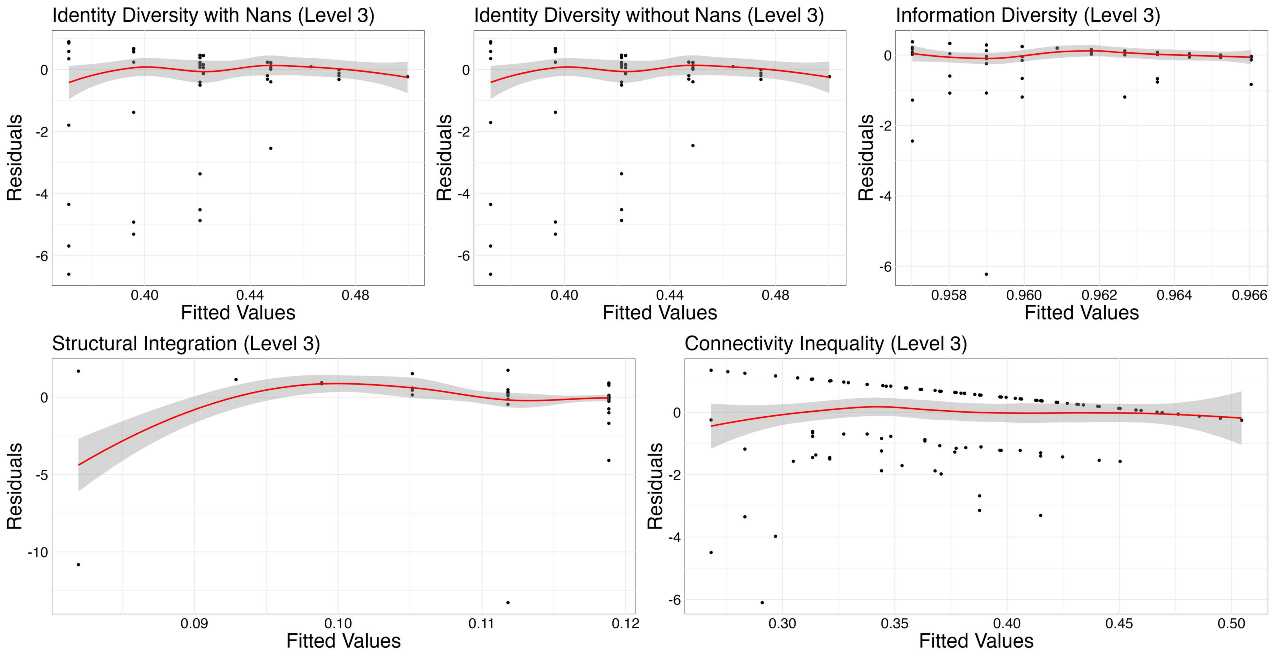

Fig V: Residual check of Beta regression models of five measurements: Identity Diversity with unlabeled accounts, Identity Diversity without unlabeled accounts, Information Diversity, Structural Integration, and Connectivity Inequality, at scale level 3.

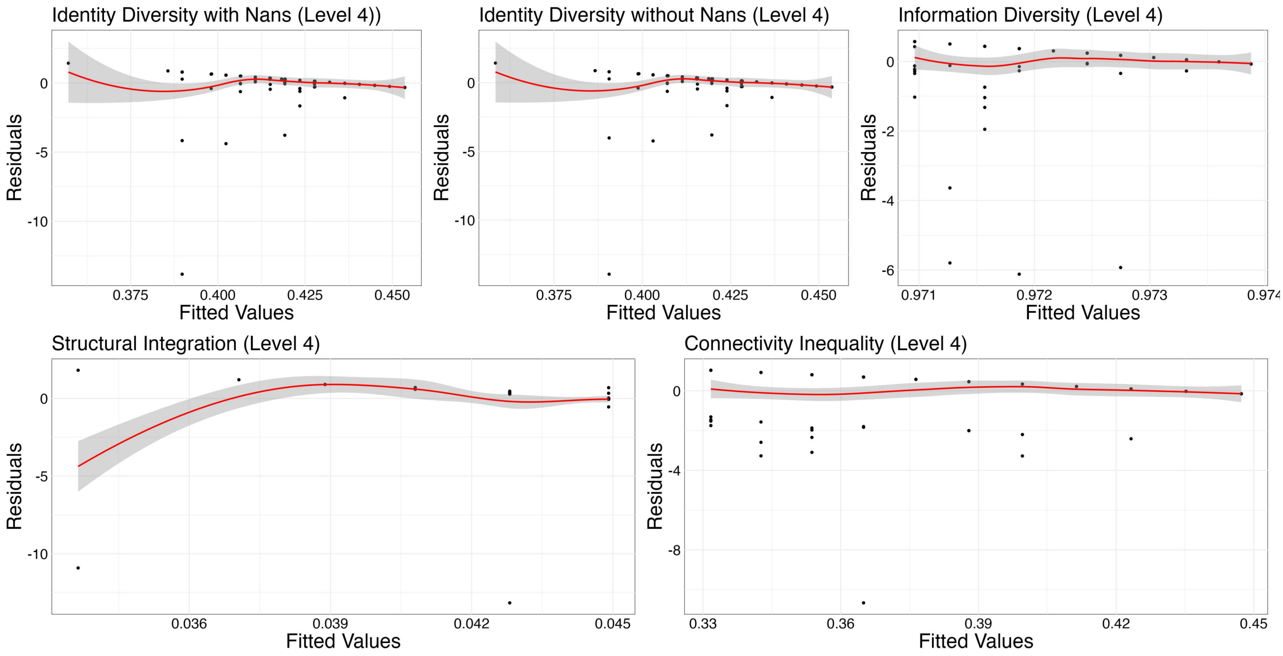

Fig W: Residual check of Beta regression models of five measurements: Identity Diversity with unlabeled accounts, Identity Diversity without unlabeled accounts, Information Diversity, Structural Integration, and Connectivity Inequality, at scale level 4.

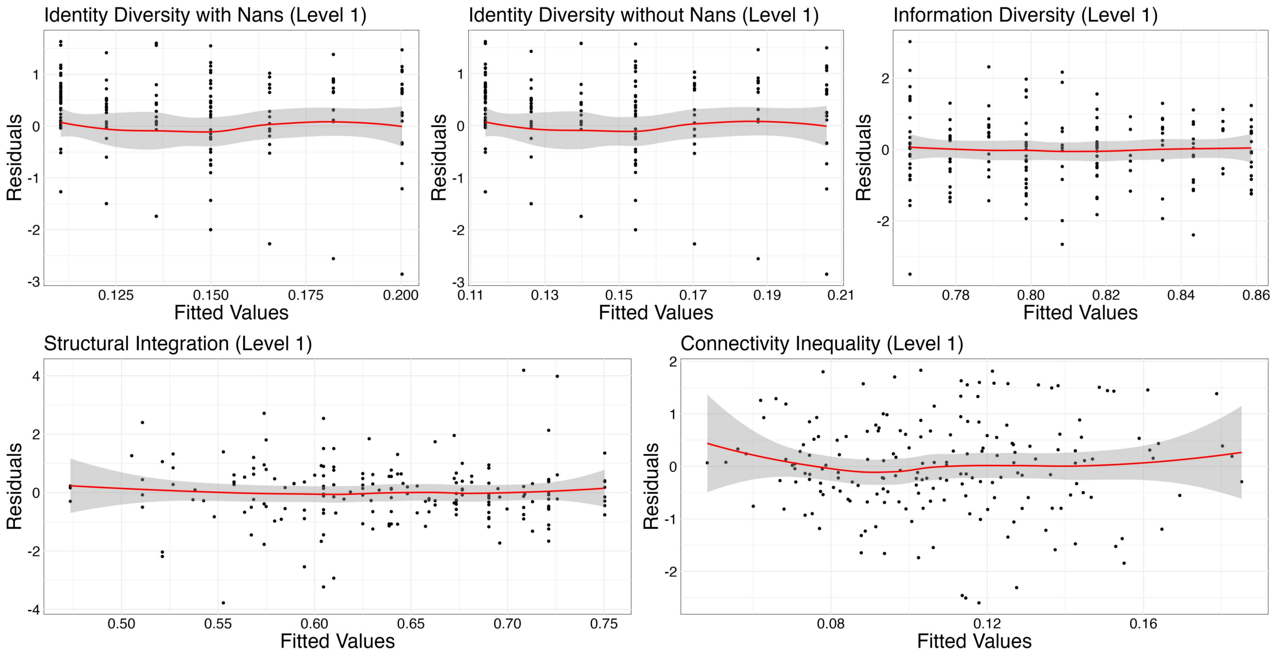

Fig X: Residual check of Beta regression models after removing outliers of five measurements: Identity Diversity with unlabeled accounts, Identity Diversity without unlabeled accounts, Information Diversity, Structural Integration, and Connectivity Inequality, at scale level 1.

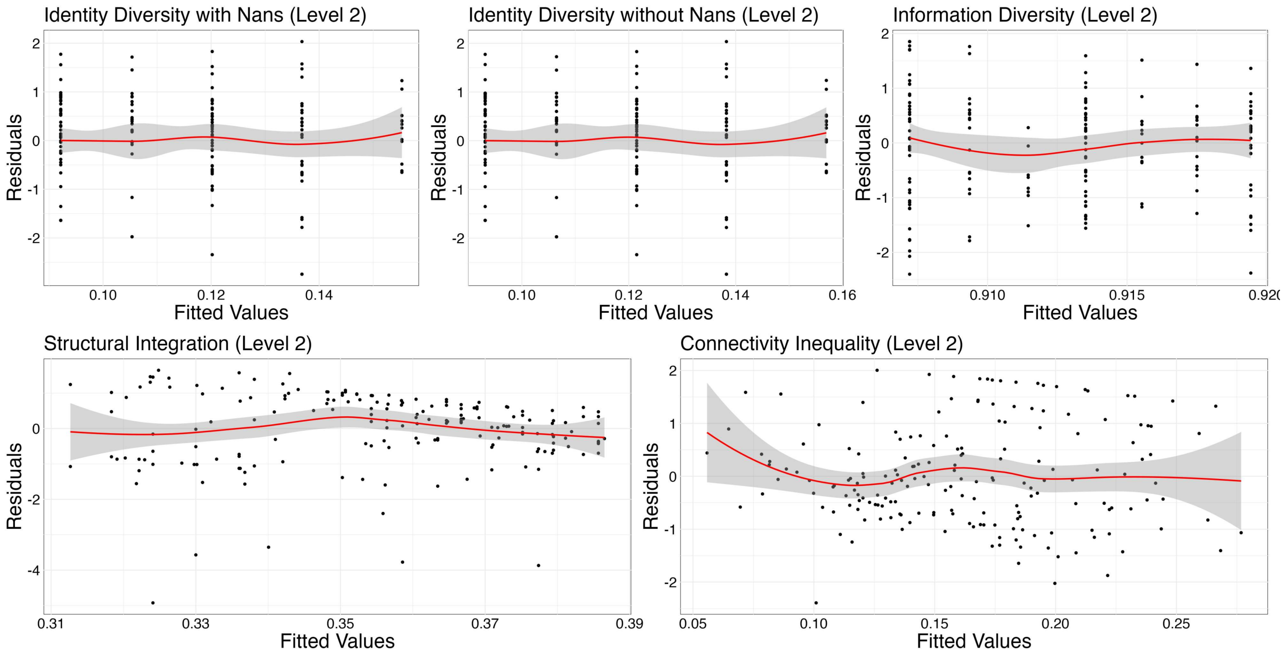

Fig Y: Residual check of Beta regression models after removing outliers of five measurements: Identity Diversity with unlabeled accounts, Identity Diversity without unlabeled accounts, Information Diversity, Structural Integration, and Connectivity Inequality, at scale level 2.

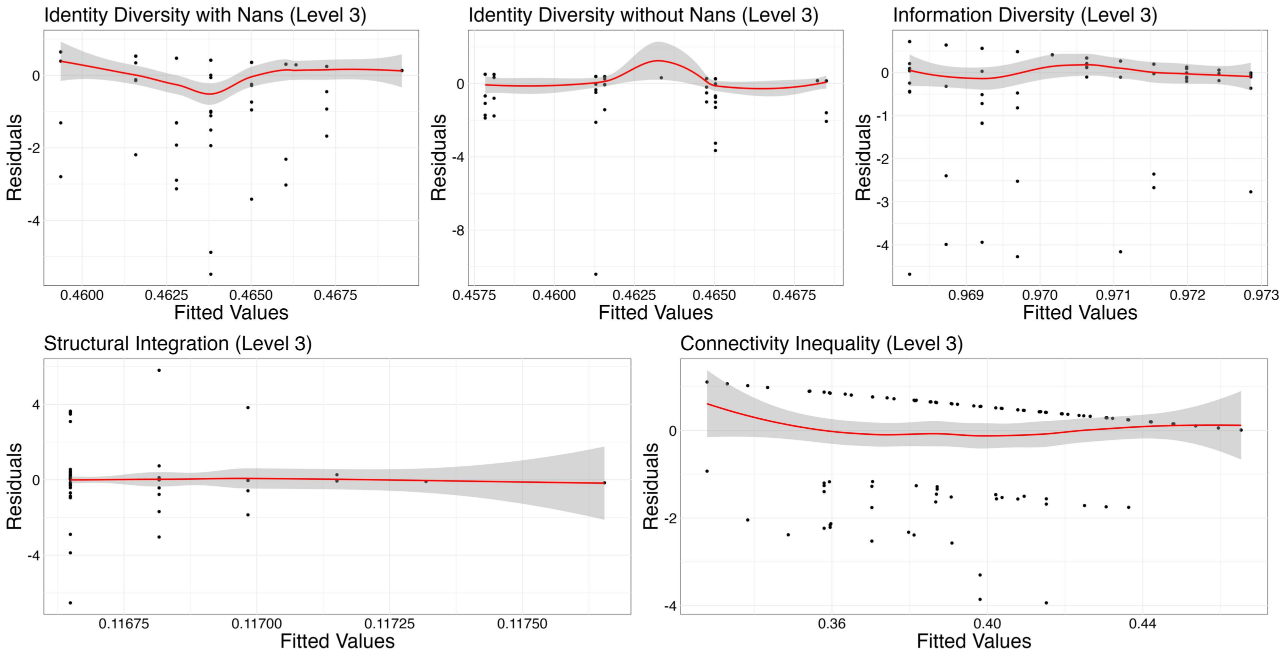

Fig Z: Residual check of Beta regression models after removing outliers of five measurements: Identity Diversity with unlabeled accounts, Identity Diversity without unlabeled accounts, Information Diversity, Structural Integration, and Connectivity Inequality, at scale level 3.

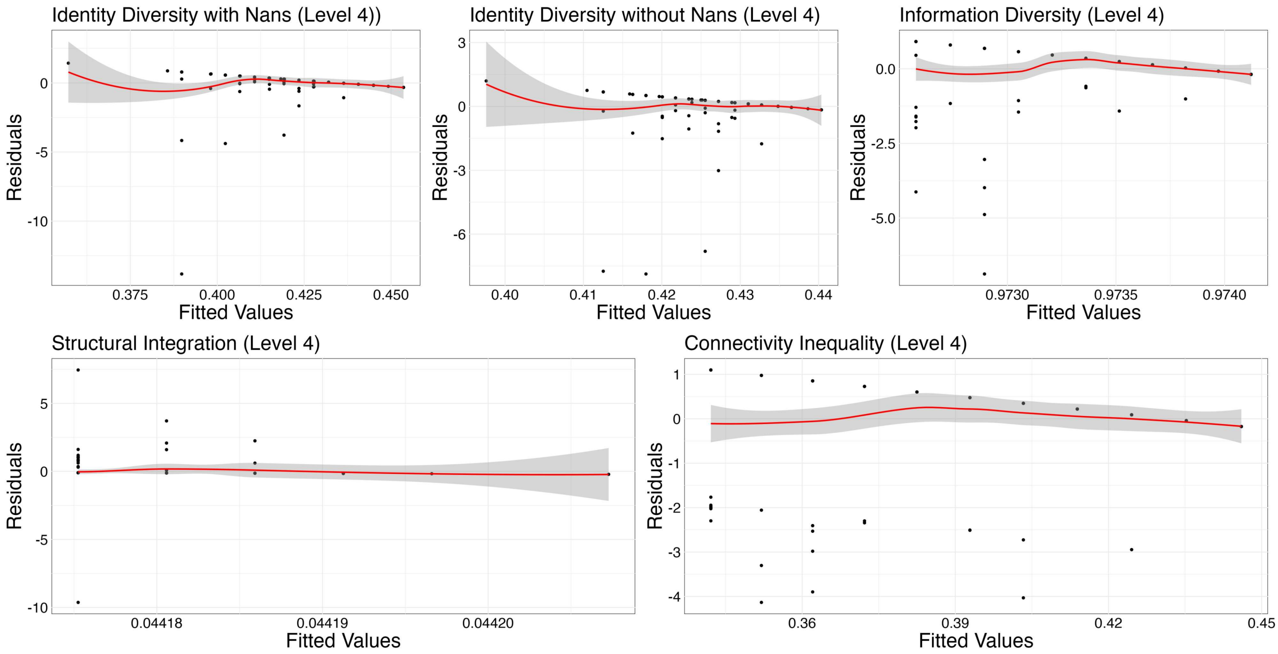

Fig AA: Residual check of Beta regression models after removing outliers of five measurements: Identity Diversity with unlabeled accounts, Identity Diversity without unlabeled accounts, Information Diversity, Structural Integration, and Connectivity Inequality, at scale level 4.

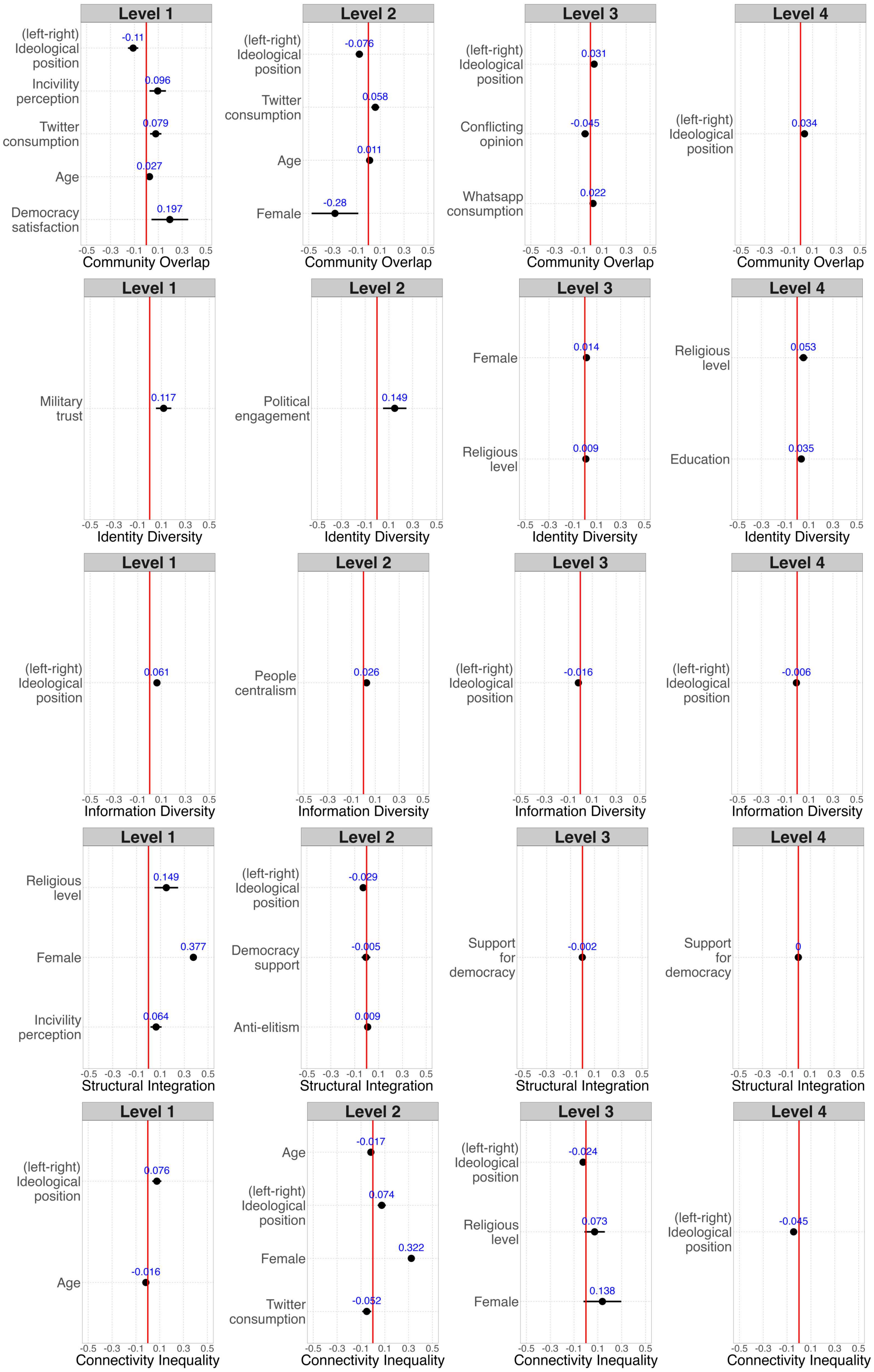

Fig AB: Regression plot of sensitivity check. Regression analysis is conducted for the five indices - Community Overlap, Identity Diversity, Information Diversity, Structural Integration, and Connectivity Inequality (only the result of the probability assignment approach for Identity Diversity Index is shown) and at four scale levels, after removing outliers in residual plots. The regression model of Structural Integration Index at scale level 4 does not converge when outliers are removed.

Table A: Soft quota of the national population provided by NetQuest.

**Variable**	**Category**	**Proportion (%)**
Gender	Hombre (Male)	49%
	Mujer (Female)	51%
Age	16–24	10%
	25–34	37%
	35–44	26%
	45–54	16%
	≥55	11%
Geographic Area	Área I	21%
	Área II	14%
	Área III	8%
	Área IV	15%
	Área V	13%
	Área VI	17%
	Área VII	9%
	Cap Norte	3%

Table B: Demographic distribution of survey participants (N = 1,018).

Variable	Category	Count	Proportion (%)
Age	16–24	102	10.0%
	25–34	370	36.3%
	35–44	269	26.4%
	45–54	164	16.1%
	55+	113	11.1%
Gender	Homem (Male)	498	48.9%
	Mulher (Female)	520	51.1%
Ethnic	Branca (White)	546	53.7%
	Preta (Black)	82	8.1%
	Parda (Mixed)	355	34.9%
	Amarela (Asian)	28	2.8%
	Indígena (Indigenous)	4	0.4%
	Outro (Other)	1	0.1%
Religion	Católico (Catholic)	351	36.4%
	P. Ev. (Prot. Evang.)	195	20.2%
	P. N-Ev. (Prot. Non-Evang.)	16	1.7%
	N-Crist. (Non-Christian)	3	0.3%
	T.J. (Jeova's Witness)	12	1.2%
	Afro-Br. (Afro-Brazilian)	36	3.7%
	Kardecista (Kardecist)	64	6.6%
	Judeu (Jewish)	6	0.6%
	Outras (Others)	30	3.1%
	Agnóstico (Agnostic)	197	20.5%
	Ateu (Atheist)	53	5.5%
Income	≤$1.2K	74	7.7%
	$1.2–2.4K	184	19.2%
	$2.4–3.6K	197	20.6%
	$3.6–6K	239	25.0%
	$6K–12K	184	19.2%
	$12–24K	55	5.7%
	$24–36K	18	1.9%
	>$36K	6	0.6%
Education	Pre-K	1	0.1%
	Elem-5	5	0.5%
	Elem-9	15	1.5%
	HS-1	12	1.2%
	HS-2	9	0.9%
	HS-3	249	24.5%
	Inc.HE	180	17.7%
	Comp.HE	345	33.9%
	PG/MSc	197	19.4%
	Ph.D.	5	0.5%

Table C: Identification of Political Influencers

Criteria	Category	Keywords
**Political Keywords**	General	política, político, political, politics, democracia, democracy
	Election	bolsonaro, bolsonarista, lula, lulista, candidato, partido, presidente
	Public sector	federal, conselho nacional de, ministro, senador, deputado, governador, prefeito, vereador, secretário
	Ideology	conservador, conservative, liberal, liberalismo, libertairia, esquerdopata, esquerda, direita, direitista, comunista, comunismo, nacionalista, patriota, globalista, feminista, armamentista, fascista, racist, colonialista, socialista, ativista, progressista
	Topic (culture)	aborto, mulher, preta, lgbt, gay, bissexualismo, homophobic, catílico, jesus, deus, ambiente, clima, justiça, imigrante, foreigner
	Topic (economic)	economia, bem-estar, pobre, desigualdade
**Political Accounts**	Political party	Partido da Mulher Brasileira, Partido dos Trabalhadores, Partido da Social Democracia Brasileira, Progressistas, Partido Democrático Trabalhista, Partido Trabalhista Brasileiro, União Brasil, Partido Liberal, Partido Socialista Brasileiro, Republicanos, Cidadania, Partido Comunista do Brasil, Partido Social Cristão, Podemos, Partido Social Democrático, Partido Verde, Patriota, Solidariedade, Partido da Mobilização Nacional, Avante, Partido Trabalhista Cristão, Partido Socialismo e Liberdade, Democracia Cristã, Partido Renovador, Trabalhista Brasileiro, Partido Republicano da Ordem Social, Partido da Mulher Brasileira, Partido Novo, Rede Sustentabilidade, Partido Socialista dos Trabalhadores Unificado, Partido Comunista Brasileiro, Partido da Causa Operária, Unidade Popular, Avante, Agir, MDB Nacional
	Politician	Aldo Rebelo, Soraya Thronicke, Jair Bolsonaro, Luiz Inácio Lula da Silva, Ciro Gomes, Simone Tebet, André Janones, Luiz Felipe D'Avila, José Maria Eymael, Leonardo Péricles, Sofia Manzano, Vera Lúcia Salgado, Luciano Bivar, Pablo Marçal, Wilson Witzel, Janaina Paschoal, José Reguff, Ibaneis Rocha, Renan Filho, Renato Casagrande, Michel Temer, Jorge Kajuru, Padre Kelmon
**Media Keywords**	Individual aggregator	jornalista, journalist, correspondent, repórter, comandante, commentator, comentarista, influencer, news, semanal
**Media Accounts**	News outlet	Globo News online (incl. G1), UOL online, Record News online (incl. R7.com), O Globo online, Band News online, Folha de S. Paulo online, O Estado de S. Paulo online, BBC News online, Rede TV News online, notícias, Jornal Extra online, TV SBT (incl. SBT Brasil), TV Band News, CNN, TV Brasil (public broadcaster)

Table D: Qualitative interpretation of sub-dimensions of independent variables reflected by PCA projection.

**Defined Groups**	**Sub-dimensions**	**Interpretation**
Demographics	Demographics	Age, Religion, Religious level, Gender, Ethnic, Education, Living, Income
News Consumption	Information from news	Frequency of news consumption from online sources such as Television, Social Media, and other digital sources
	Information from weak ties	Whether they receive political information from people they don't know very well (e.g., colleagues, acquaintances, neighbors)
	Information from strong ties	Whether they receive political information from family and friends
Political Communication	Discussion conflict	To what extent they enjoy political discussions and conflicts
	Opinion Alignment	Frequency of encountering different political opinions
Political Identification	Ideological position	Ideological spectrum from Left to Right
	Partisan degree	To what extent they feel aligned with a political party
Political Engagement	Political Engagement	Frequency of engaging in political activities, such as electoral campaign, political protest, civic activities, and online discussions
Incivility Perception	Online incivility perception	To what extent they perceive uncivil discourses on social media
	Online-Offline incivility comparison	To what extent they perceive uncivil discourses on social media compared to offline experience
Disinformation Perception	Online disinformation perception	To what extent they perceive disinformation on social media
Authority Trust	Political Trust	To what extend they trust the parliament, politicians, political parties, media, and legal system
	Military Trust	To what extend they trust the police, military, and government
Populism	Anti-elitism	To what extent they agree that political and economic elites are harming people's interests
	People centralism	To what extent they agree that the majority of the people should be prioritized
Attitudes to Democracy	Attitudes to democracy	Support for Democracy, Satisfaction with Democracy

Table E: Results of variable selection

Sub-dimensions	Variables	Selected Variable
Demographics	Age, Religion, Religious level, Gender, Ethnic, Education, Living, Income	Age, Religion, Religious level, Gender, Ethnic, Education, Living, Income
Information from News	News_Television news, News_Online news sources, News_News via social media, Campaign News_Television news, Campaign News_Online news sources, Campaign News_News via social media, Social Media_Twitter, Read/Watch_Twitter	Read/Watch_Twitter
Information from Weak-ties	News_National newspapers, News_Regional newspapers, News_Radio news, Social Media_Facebook, 'Social Media_YouTube, Social Media_Whatsapp, Social Media_Telegram, Campaign News_National newspapers, Campaign News_Regional newspapers, Campaign News_Radio news, Read/Watch_Youtube, Read/Watch_Facebook, Read/Watch_Whatsapp, Read/Watch_Telegram, Share/Liked_Twitter, Share/Liked_Youtube, Share/Liked_Facebook, Share/Liked_Whatsapp, Share/Liked_Telegram, Comment/Post_Twitter, Comment/Post_Youtube, Comment/Post_Facebook, Comment/Post_Whatsapp, Comment/Post_Telegram, Mess Apps Information_2, Mess Apps Information_3, Mess Apps Discussion_2, Mess Apps Discussion_3, Mess App Groups	Read/Watch_Whatsapp
Information from Strong-ties	Mess Apps Information_1, Mess Apps Information_97, Mess Apps Discussion_1, Mess Apps Discussion_97	Mess Apps Information_1
Discussion Conflict	Fatigue_1, Fatigue_2, Fatigue_3, Conflict Orientation_1, Conflict Orientation_2, Conflict Orientation_3, Conflict Orientation_4, Conflict Orientation_5, Discussion_1, Discussion_2, Discussion_3, Discussion_4	Fatigue_1
Opinion Alignment	Conflicting Opinion_1, Conflicting Opinion_2, Conflicting Opinion_3, Conflicting Opinion_4	Conflicting Opinion_1

Continued on next page

Table E: (continued)

Sub-dimensions	Variables	Selected Variable
Ideological Position	Ideological Position, Politician Likeability_5, Politician Likeability_2, Politician Likeability_11, Party Likeability_2, Party Likeability_5	Ideological Position
Partisan Degree	Party Alignment, Alignment Measurement, Politician Likeability_1, Politician Likeability_3, Politician Likeability_4, Politician Likeability_6, Politician Likeability_7, Politician Likeability_8, Politician Likeability_9, Politician Likeability_10, Politician Likeability_12, Party Likeability_1, Party Likeability_3, Party Likeability_4, Party Likeability_6, Party Likeability_7, Party Likeability_8, Party Likeability_9, Party Likeability_10, Party Likeability_11, Party Likeability_12	Alignment Measurement
Political Engagement	Participation_Institutional/Electoral campaign_1, Participation_Institutional/Electoral campaign_2, Participation_Institutional/Electoral campaign_3, Participation_Institutional/Electoral campaign_4, Participation_Institutional/Electoral campaign_5, Participation_Institutional/Electoral campaign_6, Participation_Institutional/Electoral campaign_7, Participation_Protest_1, Participation_Protest_2, Participation_Protest_3, Participation_Protest_4, Participation_Civic engagement_1, Participation_Civic engagement_2, Participation_Online participation_1, Participation_Online participation_2, Participation_Online participation_3, Participation_Online participation_4, Participation_Online participation_5, Political Interest, Politician/Party Accounts Following_Twitter, Politician/Party Accounts Following_Facebook, Distance of Following Accounts	Participation_Protest_1

Continued on next page

Table E: (continued)

Sub-dimensions	Variables	Selected Variable
Online Incivility Perception	Incivility Perception_Impoliteness_Twitter, Incivility Perception_Impoliteness_Youtube, Incivility Perception_Impoliteness_Facebook, Incivility Perception_Impoliteness_Whatsapp, Incivility Perception_Impoliteness_Telegram, Incivility Perception_Physical harm/violence_Twitter, Incivility Perception_Physical harm/violence_Youtube, Incivility Perception_Physical harm/violence_Facebook, Incivility Perception_Physical harm/violence_Whatsapp, Incivility Perception_Physical harm/violence_Telegram, Incivility Perception_Negativity_Twitter, Incivility Perception_Negativity_Youtube, Incivility Perception_Negativity_Facebook, Incivility Perception_Negativity_Whatsapp, Incivility Perception_Negativity_Telegram, Incivility Perception_Personal attack_Twitter, Incivility Perception_Personal attack_Youtube, Incivility Perception_Personal attack_Facebook, Incivility Perception_Personal attack_Whatsapp, Incivility Perception_Personal attack_Telegram, Incivility Perception_Stereotype/Hate speech/Discrimination_Twitter, Incivility Perception_Stereotype/Hate speech/Discrimination_Youtube, Incivility Perception_Stereotype/Hate speech/Discrimination_Facebook, Incivility Perception_Stereotype/Hate speech/Discrimination_Whatsapp, Incivility Perception_Stereotype/Hate speech/Discrimination_Telegram, Incivility Perception_Threat to democratic freedoms_Twitter, Incivility Perception_Threat to democratic freedoms_Youtube, Incivility Perception_Threat to democratic freedoms_Facebook, Incivility Perception_Threat to democratic freedoms_Whatsapp, Incivility Perception_Threat to democratic freedoms_Telegram, Experience_1, Experience_2, Experience_3, Experience_4	Incivility Perception_Impoliteness_Twitter

Continued on next page

Table E: (continued)

**Sub-dimensions**	**Variables**	**Selected Variable**
Online-Offline Incivility Comparison	Online-offline Incivility_1, Online-offline Incivility_2, Online-offline Incivility_3, Online-offline Incivility_4, Online-offline Incivility_5, Online-offline Incivility_6	Online-offline Incivility_1
Online Disinformation Perception	False Information_Facebook, False Information_Twitter, False Information_Youtube, False Information_Whatsapp, False Information_Telegram, False Information_News Media	False Information_Youtube
Political Trust	Trust_1, Trust_2, Trust_3, Trust_4, Trust_5	Trust_3
Military Trust	Trust_6, Trust_7, Trust_8	Trust_7
Anti-elitism	Populism_1, Populism_2, Populism_4, Populism_6	Populism_1
People centralism	Populism_3, Populism_5, Populism_7	Populism_7
Attitudes to Democracy	Support for Democracy, Satisfaction with Democracy	Support for Democracy, Satisfaction with Democracy

References

[1] Susie Khamis, Lawrence Ang, and Raymond Welling. Self-branding, 'micro-celebrity' and the rise of social media influencers. *Celebrity Studies*, 8(2):191–208, 2017.

[2] Dennis Harff and Desirée Schmuck. Influencers as empowering agents? following political influencers, internal political efficacy and participation among youth. *Political Communication*, 40(2):147–172, 2023.

[3] Felipe Bonow Soares, Raquel Recuero, and Gabriela Zago. Influencers in polarized political networks on twitter. In *Proceedings of the 9th international conference on social media and society*, pages 168–177. ACM, July 2018.

[4] Elizabeth Dubois and Devin Gaffney. The multiple facets of influence: Identifying political influentials and opinion leaders on twitter. *American Behavioral Scientist*, 58(10):1260–1277, 2014.

[5] J Flamino, J Blackburn, T Caulfield, G Stringhini, S Zannettou, and E De Cristofaro. Political polarization of news media and influencers on twitter in the 2016 and 2020 us presidential elections. *Nature Human Behaviour*, pages 1–13, 2023.

[6] A. Arnaudon, D. J. Schindler, R. L. Peach, A. Gosztolai, M. Hodges, M. T. Schaub, and M. Barahona. Pygenstability: Multiscale community detection with generalized markov stability. 2023.

[7] R Lambiotte, J. C Delvenne, and M Barahona. Random walks, markov processes and the multiscale modular organization of complex networks. *IEEE Transactions on Network Science and Engineering*, 1(2):76–90, 2014.

[8] Alexis Arnaudon, Dominik J Schindler, Robert L Peach, Adam Gosztolai, Maxwell Hodges, Michael T Schaub, and Mauricio Barahona. Algorithm xxx: Pygenstability, a multiscale community detection with generalized markov stability. *ACM Transactions on Mathematical Software*, page 3651225, March 2024.

[9] Michael Smithson and Jay Verkuilen. A better lemon squeezer? maximum-likelihood regression with beta-distributed dependent variables. *Psychological Methods*, 11(1):54, 2006.

[10] Francisco Cribari-Neto and Achim Zeileis. Beta regression in r. *Journal of statistical software*, 34:1–24, 2010.

S1 Text. Survey questionnaire, additional methods, and supplementary results.
